# Deployable 3D‐Printed Vascular Stent with Surface‐Catalysed Endogenous Nitric Oxide Generation

**DOI:** 10.1002/adma.202520199

**Published:** 2026-03-15

**Authors:** Kun Zhou, Zifei Han, Kang Lin, Di Wu, Siti Nur Asyura Adzlan, Qingqing Fan, Christina Cortez‐Jugo, Rona Chandrawati, Cyrille Boyer

**Affiliations:** ^1^ School of Chemical Engineering UNSW Sydney New South Wales Australia; ^2^ Australian Centre for Nanomedicine (ACN) UNSW Sydney New South Wales Australia; ^3^ Department of Chemical Engineering The University of Melbourne Parkville Victoria Australia

**Keywords:** deployable vascular stent, endogenous nitric oxide catalysis, shape‐memory material, spatiotemporal control

## Abstract

Atherosclerosis, a leading cause of heart attacks and strokes through the formation of vascular blockages, has become a major global healthcare challenge, with patient numbers expected to rise. Balloon‐based angioplasty and stenting, the most common clinical treatments, have evolved over decades with advances in material biocompatibility, biodegradability, and device design. However, they still face critical issues such as tissue damage, inflammation, and restenosis, which can lead to implant failure and necessitate repeated surgeries, severely affecting patients’ quality of life. To address the urgent need for a long‐term stable stent system, we present a deployable 3D‐printed vascular stent with surface‐catalyzed endogenous nitric oxide (NO) generation (DSENO), offering a promising new direction for stent development. Unlike traditional stents, the DSENO can be deployed remotely using magnetic force and heat, eliminating the need for a catheter or balloon and thereby reducing the risk of tissue injury. Furthermore, its surface is modified to catalyze intracellular NO generation from endogenous S‐nitrosothiols, which inhibits smooth muscle cell proliferation to prevent restenosis.

## Graphical abstract

A deployable 3D‐printed vascular stent was fabricated with surface‐catalyzed endogenous nitric oxide generation, representing a promising new direction for stent development. The stent can be remotely controlled by magnetic force in its compressed state and fully deployed at the target site using an untethered heat source, potentially eliminating the need for a catheter or balloon and thereby reducing the risk of tissue injury.

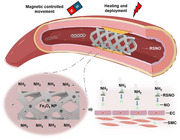

## 1 Introduction

Cardiovascular diseases are the leading cause of death worldwide, accounting for 19.8 million deaths—about 32% of all global deaths in 2022 [1]. Atherosclerosis, the underlying cause of many cardiovascular conditions, triggers heart attacks and strokes by obstructing blood vessels [2]. This blockage results from the progressive accumulation of lipids and platelets [3] on vessel walls [4], leading to thickening, narrowing, and restricted blood flow [5, 6]. To maintain adequate perfusion, blood pressure increases, which can accelerate vascular calcification [2, 4, 7]. Calcification, in turn, increases vessel stiffness, promoting further lipid and platelet deposition—a vicious cycle that exacerbates disease progression [8]. Although the severity of atherosclerosis is well recognized, its rising incidence among young adults [9] and patients in developing countries [10] is concerning, driven by inadequate early screening in the former and limited access to diagnostic and treatment devices in the latter [11, 12].

The demand for effective atherosclerosis treatments is therefore expected to grow substantially. Current options include coronary artery bypass grafting, angioplasty, and stenting, with angioplasty and stenting often performed together [13]. Angioplasty widens narrowed or blocked vessels via balloon inflation, and the subsequent placement of a stent maintains restored blood flow while reducing the risk of acute thrombosis and restenosis [14, 15, 16]. Over decades, cylindrical stent designs have evolved from bare‐metal stents (BMS) [17] to drug‐eluting stents (DES) [18, 19, 20, 21] and bioresorbable stents (BRS) [22, 23, 24]. Compared with BMS, DES incorporates a polymer coating loaded with drugs, where the polymer controls release either via diffusion (non‐degradable) [25] or degradation (biodegradable) [26]. DES significantly reduces early restenosis and delays recurrence, but once the drug is fully released, the permanent implant can still cause late restenosis [27, 28, 29]. To overcome this limitation, BRS was introduced with the concept of complete absorption after the treatment period, theoretically eliminating delayed restenosis [30, 31, 32, 33].

However, significant challenges remain for both angioplasty and stenting. In angioplasty, the catheter delivers the balloon to the target site for inflation, but the procedure can damage the arterial wall and surrounding tissues, triggering inflammatory responses that increase the risk of restenosis [33, 34, 35]. For stenting, beyond the limitations of BMS and DES, BRS faces clinical issues that undermine their theoretical advantages. Incomplete resorption can release acidic degradation products [36, 37], provoking severe inflammation [38, 39], while the loss of structural integrity [40, 41] and suboptimal degradation profiles may compromise early vessel scaffolding [42, 43]. These factors have resulted in non‐advanced clinical performance of BRS compared to DES [44, 45, 46]. Addressing these limitations demands an advanced stent system capable of minimizing inflammatory responses and preventing late‐stage restenosis.

**FIGURE 1:**
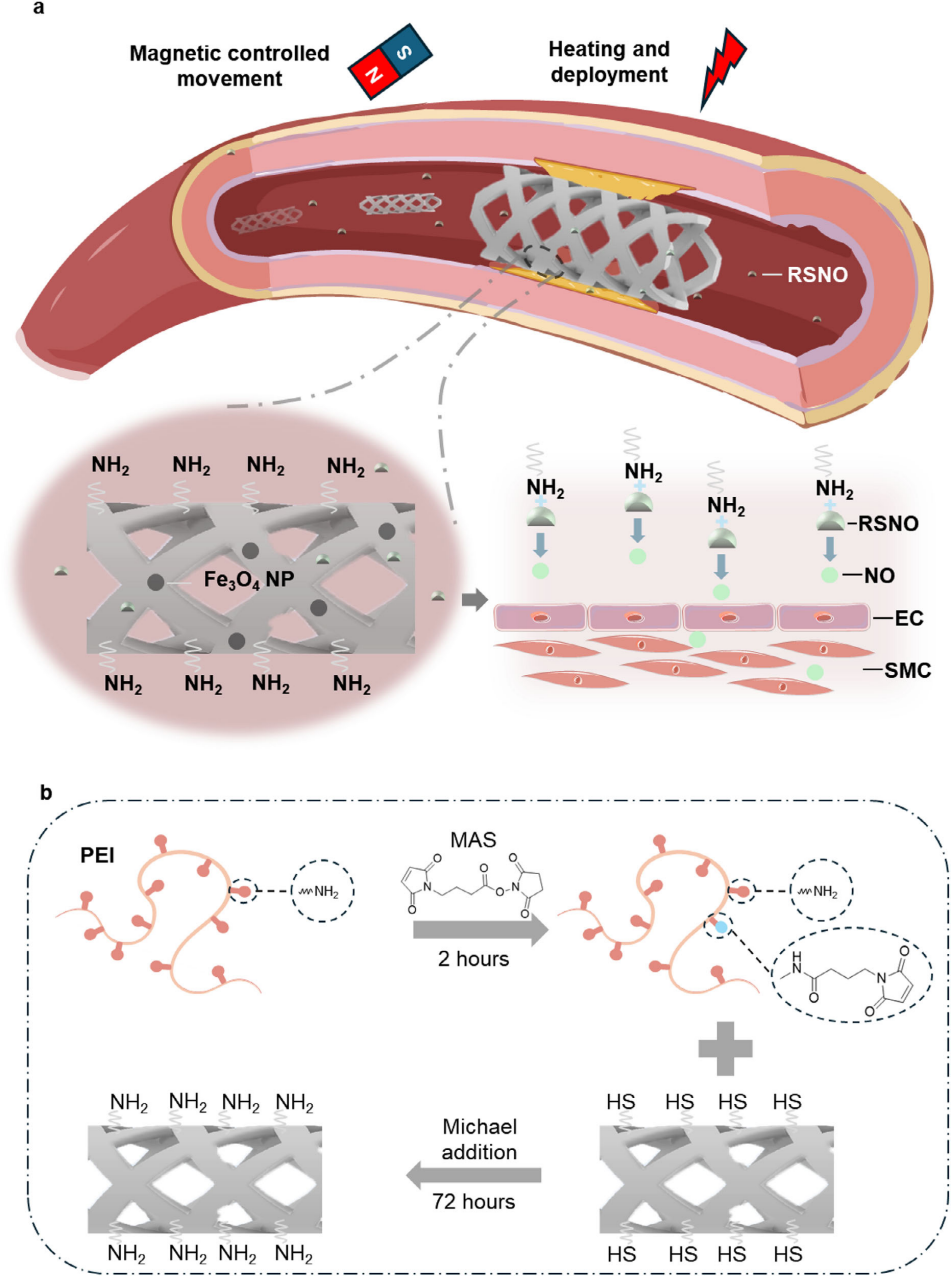
Schematics of DSENO working mechanism and surface modification with PEI coating. (a) Working mechanism: The stent, in a compressed state, is magnetically guided to the treatment site. Upon localized heating, it expands and recovers its original shape, providing structural support to maintain vessel patency. The stent surface is coated with polyethyleneimine (PEI), a polymer rich in primary amine groups (–NH_2_), which catalyse the decomposition of endogenous S‐nitrosothiols (RSNOs) naturally present in the bloodstream. This catalytic reaction continuously generates nitric oxide (NO) directly on the stent's surface, thereby inhibiting smooth muscle cells (SMCs) proliferation and preventing restenosis. (b) PEI coating process: Schematic illustration of PEI deposition on the stent surface to introduce catalytic amine groups.

Herein, we report a deployable 3D‐printed vascular stent, termed DSENO, that integrates shape‐memory functionality with surface‐catalyzed endogenous nitric oxide (NO) generation. By leveraging high‐resolution 3D printing, DSENO allows for the fabrication of patient‐specific architectures precisely tailored to individual vascular anatomies. This level of personalization is critical to ensuring optimal wall apposition, thereby mitigating common clinical complications such as stent migration or incomplete expansion. The structural core of the DSENO consists of a shape‐memory polymer (SMP) composite embedded with magnetic Fe_3_O_4_ nanoparticles, which facilitates remote, untethered deployment via magneto‐thermal activation (Figure 1a). Unlike traditional balloon angioplasty, this magnetically guided approach bypasses the need for bulky catheters, significantly reducing procedural tissue trauma and the subsequent inflammatory response [47]. In contrast to bioresorbable scaffolds (BRS), which are often hindered by unpredictable degradation kinetics and the release of pro‐inflammatory acidic by‐products, DSENO is engineered as a non‐degradable, permanent implant that provides stable, long‐term mechanical scaffolding. The stent surface is functionalized with polyethyleneimine (PEI), whose amine groups catalyze the decomposition of endogenous S‐nitrosothiols (RSNOs) for sustained, in situ NO release (Figure 1a,b). While drug‐eluting stents (DES) are limited by finite reservoirs [48] and late‐stage restenosis post‐depletion [49, 50, 51, 52], the DSENO's catalytic mechanism ensures sustained NO production. This dual‐action surface simultaneously promotes endothelialization and inhibits smooth muscle cell (SMC) proliferation, effectively preventing restenosis. Collectively, these features position the DSENO as a transformative alternative to current BRS and DES technologies, offering a permanent, bio‐instructive solution for vascular intervention. In this study, we detail the optimization of the printing resin, material characterization, surface modification, and functional performance, confirming the feasibility of the DSENO. As a permanent stent with continuous endogenous NO generation, the DSENO represents a promising new direction in vascular stent technology, capable of offering a long‐term solution to a major global healthcare challenge.

## 2 Results and Discussion

### 2.1 Resin Optimization of Thermal Properties

**FIGURE 2:**
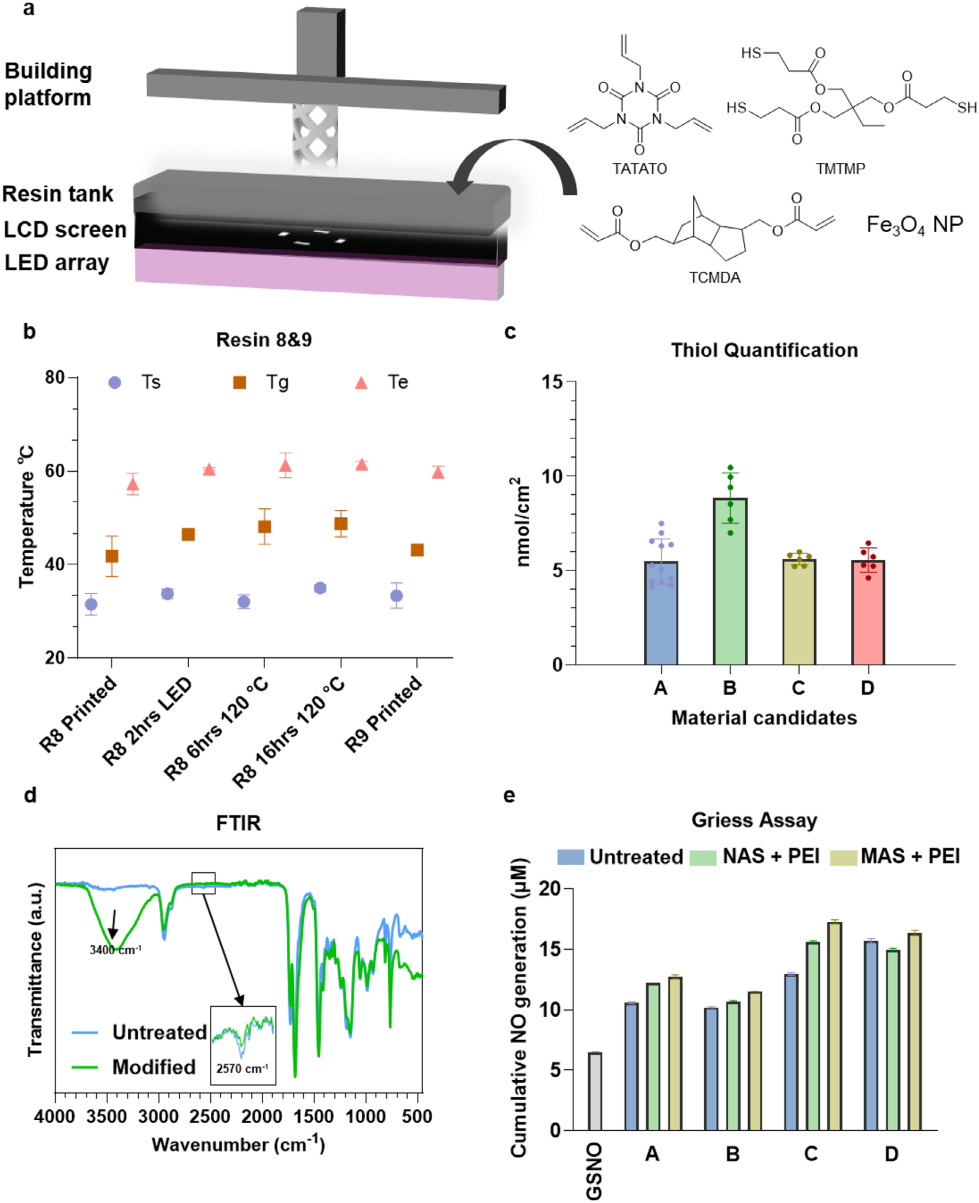
Surface modification and characterizations of 3D‐printed surface before and after functionalization. (a) Printing method and resin composition. (b) The T_s_, T_g_, and T_e_ of materials printed with resin 8 and 9 after different post‐treatments. *n* = 3. (c) Quantified surface thiol densities of four material candidates. Material A: printed with Resin 9 (no post‐treatment). Material B: printed with Resin 8 (no post‐treatment). Material C: printed with Resin 8 followed by 2 h post‐curing. Material D: printed with Resin 8 followed by 6 h heating at 120°C. (d) FTIR spectra of material A before and after PEI coating. (e) Cumulative NO generation comparison among the four material candidates.

The DSENO was fabricated using an LCD 3D printer with a customized resin formulation containing three crosslinkers, specifically trimethylolpropane tris(3‐mercaptopropionate) (TMTMP), tricyclo[5.2.1.02,6] decanedimethanol diacrylate (TCMDA), and 1,3,5–triallyl‐1,3,5‐triazine‐2,4,6(1H,3H,5H)‐trione (TATATO), to form a shape‐memory polymer network (Figure 2a) [53]. TMTMTP introduced the thiol group for the following surface coating, TCMDA performed as a rigid linker, and TATATO tuned the material flexibility by staged crosslinking. Besides, Fe_3_O_4_ nanoparticles were incorporated into this network to enable stent untethered delivery by magnetic control in the compressed state and deployment at the treatment location via remote heating (Figure 2a). To achieve the proposed functions, the printing resins needed to be screened for optimal material thermal properties by varying crosslinker and nanoparticle weight ratios (Table S1). It was predictable that the crosslinked shape‐memory polymer network often exhibits a broad glass transition temperature (*T*_g_) range. To prevent premature deployment during transportation, the glass transition starting temperature (*T*_s_) should exceed 37°C, and *T*_g_ would need to be significantly above physiological temperature. Both *T*_s_ and *T*_g_ were determined by differential scanning calorimetry (DSC) (Figure S1). However, a high‐*T*_g_ increases the risk of vessel tissue burns and severe inflammation during the deployment, which could trigger acute restenosis. Therefore, during material screening, the optimal criteria were set as: *T*_s_ higher than room temperature but close to 37°C, and *T*_g_ above 37°C and below 50°C. These parameters would allow DSENO to remain relatively stable after implantation and then either rapidly deploy upon heating at the target site or slowly recover without external heating.

In the first five resins (resin #1–5), the content of TMTMP was systematically increased. For resin 2–3, the increase of TMTMP led to a higher *T*_g_ value of resin 2 but a lower *T*_g_ value for resin 3 (Figure S2). Thermal post‐processing enhanced *T*_g_, with longer durations and higher temperatures producing stronger effects; in particular, post‐curing at 120°C proved more effective than at 70°C for resins 1–3 (Figure S2), resulting in a significant increase in *T*_g_. Post‐curing with LED light (405 nm) also significantly increased *T*_g_, although its penetration was limited, especially in black‐pigmented materials containing Fe_3_O_4_ nanoparticles, resulting in greater network heterogeneity. This heterogeneity was reflected in the DSC curves as a larger gap between the glass transition onset (*T*_s_) and end (*T*_e_) temperatures in light‐cured samples (Figures S1 and S2). Long‐term light post‐curing could significantly increase *T*_g_, but this method raised concerns that residual surface thiols required for subsequent postmodification might be depleted. Therefore, a combined strategy involving long‐term thermal treatment followed by a short‐term light post‐curing was adopted, which improved network homogeneity and increased *T*_g_, although the values still remained below the target range.

Further DSC analysis of resins 4–5 revealed that increasing the amount of TMTMP improved network homogeneity but, critically, decreased the *T*_g_. Attempts to recover this *T*_g_ loss by increasing post‐treatment time yielded only minor improvements (Figure S2d). To better balance homogeneity and thermal properties, resin 6 was formulated based on resin 4 by doubling the concentration of TATATO and decreasing the concentration of Fe_3_O_4_. While this modification yielded good homogeneity, as evidenced by a narrow *T*_s_ – *T*_e_ gap, the *T*_g_ dropped even further (Figure S3a). These findings prompted a new strategy: increasing the amount of TCMDA, containing a rigid linker, while maintaining a constant weight ratio of TMTMP to TATATO. This approach successfully raised the *T*_g_ of the polymer network printed with resin 7 (44.45 wt.% TCMDA), bringing it closer to the required level (Figures S2b and S4), while resins 8 (54.35 wt.% TCMDA) and 9 (61.14 wt.% TCMDA) ultimately achieved the desired *T*_s_ around 33°C and *T*_g_ around 43°C (Figure 2b). Consequently, four material candidates printed with resins 8 and 9 (Table S2) were selected for subsequent surface modification, NO production assessment, and biocompatibility evaluation.

### 2.2 Surface Modification for NO Catalysis and In Vitro Characterizations

The free thiol groups present on the 3D‐printed surfaces were leveraged for surface functionalization. To validate and quantify these thiols, Ellman's reagent was employed (Figure S6a), and surface thiol density was determined using a calibration curve generated from L‐cysteine (Figure 2c; Figure S6b). Among the tested materials, material B printed with resin 8 without post‐treatment showed the highest thiol density around 7 nmol cm^−2^, attributed to its higher TMTMP content in the resin formulation and the absence of post‐processing steps that would otherwise consume thiols. While this quantification confirmed the feasibility of surface modification, direct coupling of PEI to the surface was not possible due to their chemical structures. To address this, 4‐maleimidobutyric acid *N*‐hydroxysuccinimide ester (MAS) was first reacted with PEI to yield maleimide‐functionalized PEI (Figure 1b). The resulting maleimide‐PEI was subsequently coupled to the 3D‐printed surface via Michael thiol‐ene addition.

Successful functionalization of the 3D printed surface with PEI was confirmed by Fourier‐transform infrared spectroscopy (FTIR), which shows the presence of amine bonds at 3300–3500 cm^−1^ (Figure 2d) [54]. In parallel, a slight reduction in the intensity of the thiol stretching band at approximately 2570 cm^−^^1^ was observed after PEI coating, indicating partial consumption of surface thiols during the modification process. This observation was further supported by quantitative thiol analysis using Ellman's assay, which confirmed thiol consumption after PEI coating (Figure S7). Since direct quantification of PEI surface density was not feasible, catalytic performance was assessed directly using the Griess assay to measure NO release from the decomposition of S‐nitrosoglutathione (GSNO), an endogenous NO donor (Figure 2e). For these tests, GSNO self‐decomposition and untreated materials served as negative controls, while PEI‐treated samples with *N*‐acryloxysuccinimide (NAS) crosslinker served as positive controls. The Griess assay revealed distinct differences among polymer formulations and crosslinkers. Across all cases, MAS outperformed NAS, highlighting the advantage of Michael addition–based linkage. Interestingly, material candidate B, which exhibited the highest surface thiol density, showed the lowest cumulative NO release over 24 h. This reduction may be due to disulfide bond formation and steric hindrance, which limit the availability of thiolates and impair PEI coating efficiency. Consequently, candidate B was excluded from further evaluation. Notably, uncoated samples also displayed catalytic activity, likely arising from Fe_3_O_4_‐induced GSNO decomposition [55]. Notably, while uncoated samples displayed baseline catalytic activity, control experiments using free Fe_3_O_4_‐nanoparticles (matching the 2 wt.% loading of the printed plates) demonstrated no enhancement in NO generation (Figure S8). This rules out the magnetic fillers as primary catalysts. Instead, the baseline activity of uncoated plates is attributed to surface‐accessible thiol groups (Figure 2c), while the robust, accelerated NO release in the functionalized samples originates predominantly from the PEI‐based catalytic surface.

**FIGURE 3:**
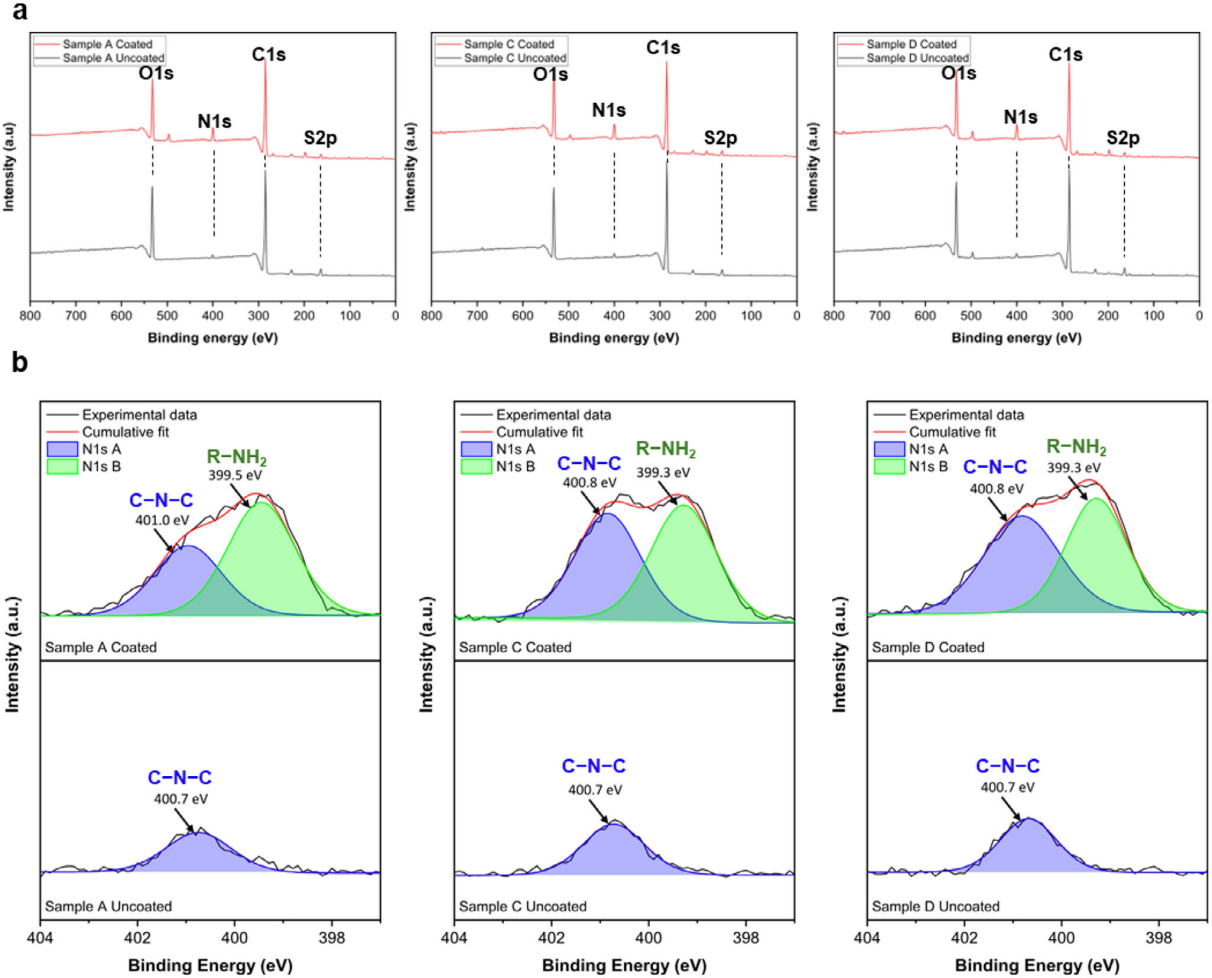
XPS analysis of coated and uncoated candidate materials A, C, and D. (a) Survey scans. (b) High‐resolution N1s spectra. Note: While the incorporation of Fe_3_O_4_ nanoparticles was confirmed through magnetic and photothermal validation, they remained undetected during XPS analysis (Figure 3a). This is attributed to the surface sensitivity of XPS (top 5–10 nm), as the bulk‐dispersed nanoparticles are shielded by the overlying polymer layer and PEI coating. The presence of Fe in the bulk material is confirmed by EDS analysis (see Figure S5).

Before biological assessment, X‐ray Photoelectron Spectroscopy (XPS) was employed to further analyze the elemental composition of DSENO materials and verify successful amine group coating (Figures 3; Figure S6). Survey scans of uncoated materials A, C, and D showed dominant peaks at ≈530, ≈280, and ≈170 eV, corresponding to oxygen, carbon, and sulfur, whereas PEI‐coated samples exhibited an additional nitrogen peak at ≈400 eV (Figure 3a) [56]. The peak positions, intensity ratios, and corresponding group assignments are summarized in Table S3. The spectrum of S2p revealed three components, and the atomic ratio decreased to < 2% after coating (Figure S9b and Table S4). High‐resolution N1s spectra revealed two distinct peaks at 399.3 and 400.8 eV for PEI‐coated DSENO materials, compared to a smaller single peak at ≈400.7 eV in uncoated samples, confirming the presence of both primary amines and maleimide–PEI coating (Figure 3b) [57, 58]. Quantitative analysis of N1s demonstrated a significant increase in nitrogen content, with the atomic ratio rising from <2% in uncoated samples to 6–7% after PEI coating (Figure S9c). Further analysis of the C1s region in pristine DSENO candidates revealed three main components: C═O (around 290.0 eV), C─N/C─O (around 286.3 eV), and C─C (around 284.8 eV). In coated groups, a new peak at 287.4 eV (C1s D) emerged, attributed to the amide bond (O = C─N) introduced by NHS ester–amine coupling (Figure S9). The corresponding O1s C peak at 531.0 eV further confirmed the presence of amide carbonyl oxygen (Table S3).

Surface wettability was evaluated by water contact angle analysis (Figure S10). Uncoated DSENO polymer surfaces were dominated by hydrophobic groups, with contact angles between 80–90°. After PEI coating, the introduction of high‐density polar amine and amide groups significantly improved hydrophilicity, reducing water contact angles to 50–70°. These results collectively proved successful PEI coating across all tested DSENO materials (A, C, and D).

**FIGURE 4:**
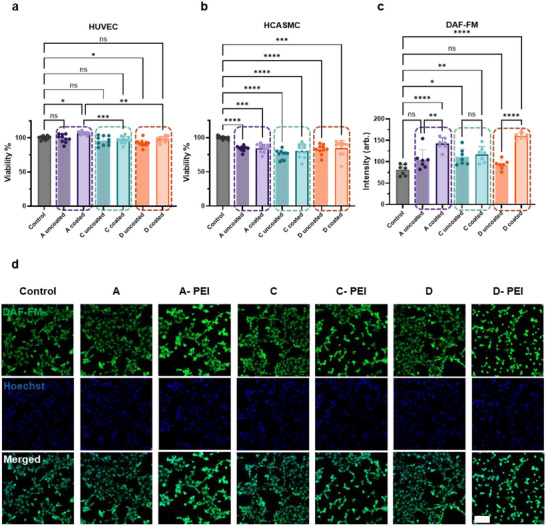
In vitro biocompatibility and intracellular NO generation characterisation of DSENO materials. (a, b) Cell viability of HUVECs (a) and HCASMCs (b) after 48 h incubation with printed DSENO plates. (c) Quantitative analysis of material‐induced intracellular NO generation. Endogenous NO production in HUVECs was quantified by measuring the mean fluorescence intensity (MFI) of the DAF‐FM probe using high‐resolution confocal laser scanning microscopy (CLSM). (d) CLSM images of HUVECs after 48 h incubation with material candidates, cells stained with DAF‐FM (green) and Hoechst (blue) (*n* ≥ 6). Scale bar = 100 µm. Data are normalized to the blank control and represent the average MFI of multiple regions of interest. Error bars indicate standard deviation; statistical significance was determined via one‐way ANOVA (^*^ *p* < 0.05).

To assess the biocompatibility of the DSENO material candidates, in vitro cellular studies were conducted using 3D‐printed plates of each candidate material, both with and without PEI coating. Human umbilical vein endothelial cells (HUVECs) and human coronary artery smooth muscle cells (HCASMCs) were selected as representative vascular cell types. These cells were chosen to evaluate the dual function of DSENO materials: suppressing smooth muscle cell proliferation through NO release, while preserving compatibility with endothelial cells. Cell viability was evaluated after 48 h of incubation with 3D printed DSENO plates using the Alamar Blue assay. Biocompatibility analysis with HUVECs revealed no significant difference in cell viability when incubated with uncoated candidates A and C, while candidate D caused a minor reduction in cell proliferation (Figure 4a). Upon PEI coating, candidates C and D showed no difference from the blank control, while PEI‐coated candidate A displayed increased HUVEC proliferation, higher than the control group. In contrast, all DSENO candidates (A, C, and D) significantly inhibited the proliferation of HCASMCs (Figure 4b). This selective inhibition is critical because aberrant SMC migration and proliferation lead to intimal hyperplasia and subsequent in‐stent restenosis, the ability of DSENO materials to regulate SMC growth is highly advantageous for long‐term stent efficacy. Furthermore, to address potential risks from unreacted or leachable resin monomers, extractable cytotoxicity testing was performed. Conditioned media, collected at multiple time points over an extended incubation period, were applied to HUVEC cultures. As shown in Figure S11, cell viability remained comparable to control groups across all extraction conditions. To verify safety under conditions more relevant to stent applications, direct‐contact assays were also conducted (Figure S12). These results confirmed no significant reduction in HUVEC viability on PEI‐coated surfaces compared to blank controls, further supporting the biological safety and robust polymerization of the 3D‐printed DSENO materials.

The intracellular NO‐generating capacity of DSENO materials was evaluated in endothelial cells, relying exclusively on endogenous NO donors without external supplementation. The HUVECs were stained with DAF‐FM diacetate, a cell‐permeant fluorescent NO probe, to compare endogenous NO release among PEI‐coated materials, uncoated candidates, and blank controls after 48 h of incubation. DAF‐FM can react with intracellular NO at low concentrations to form a fluorescent benzotriazole, and the mean fluorescence intensity was quantified using confocal microscopy to assess NO generation in each group (Figure 4c–d). The blank control group exhibited low baseline NO expression, consistent with physiological NO production from L‐arginine via endothelial nitric oxide synthase. Uncoated material candidates showed NO levels comparable to the control, whereas PEI‐coated candidates consistently exhibited higher NO release. Notably, PEI‐coated candidates A and D showed substantially elevated NO production, at 1.8‐fold and 2.0‐fold higher than the control group, respectively. As a key signaling molecule, NO regulates vascular homeostasis and vasodilation in both endothelial and smooth muscle cells. Therefore, the enhanced endogenous NO release from DSENO without an exogenous source highlights its therapeutic potential. Taken together, the combination of catalytic activity, biocompatibility, and NO‐mediated cellular effects identified PEI‐coated candidate A (derived from resin 9) as the most promising formulation, which was selected for the remainder of this study.

Hemocompatibility is a critical requirement for blood‐contacting vascular implants. Accordingly, the blood compatibility of the 3D‐printed plates was evaluated using fresh human whole blood through hemolysis and whole‐blood coagulation assays [59]. As shown in Figure S13, all uncoated and coated samples exhibited negligible hemolysis (<1%), remaining well below the threshold specified by ISO 10993–4 [60, 61], indicating minimal red blood cell damage upon direct blood contact. To further assess anticoagulant performance, whole‐blood coagulation assays were performed (Figure S14). After 10 min of incubation, thrombus formation was observed on uncoated samples, whereas coated surfaces showed minimal clot adhesion. At an extended incubation time of 15 min, thrombus formation persisted on all uncoated samples, while no visible thrombus was detected on the A‐coated surface. These results demonstrate that the NO‐generating coating effectively suppresses surface‐induced coagulation, with the A‐coated material exhibiting the most sustained anticoagulant behavior.

Beyond cell viability and hemocompatibility, the impact of the DSENO platform on endothelial function was evaluated via immunofluorescence staining of VE‐cadherin and F‐actin (Figure S15). VE‐cadherin is a critical junctional protein required for maintaining the endothelial permeability barrier and regulating intracellular signaling. Quantitative analysis of fluorescence intensity revealed that PEI‐coated material A supported healthy VE‐cadherin levels comparable to the blank control, and significantly higher than those observed for the uncoated material (Figure S15a). Notably, while HUVECs incubated with uncoated material A exhibited disrupted junctional morphology, the PEI‐coated samples maintained continuous and robust VE‐cadherin localization at cell–cell interfaces (Figure S15b). Coupled with the organized F‐actin cytoskeleton, these results demonstrate that the catalytic NO release effectively preserves endothelial junctional integrity and supports the formation of a stable, healthy endothelium—a critical prerequisite for long‐term stent application.

### 2.3 Printing Resolution and Shape‐Memory Validation of the Printing Material

**FIGURE 5:**
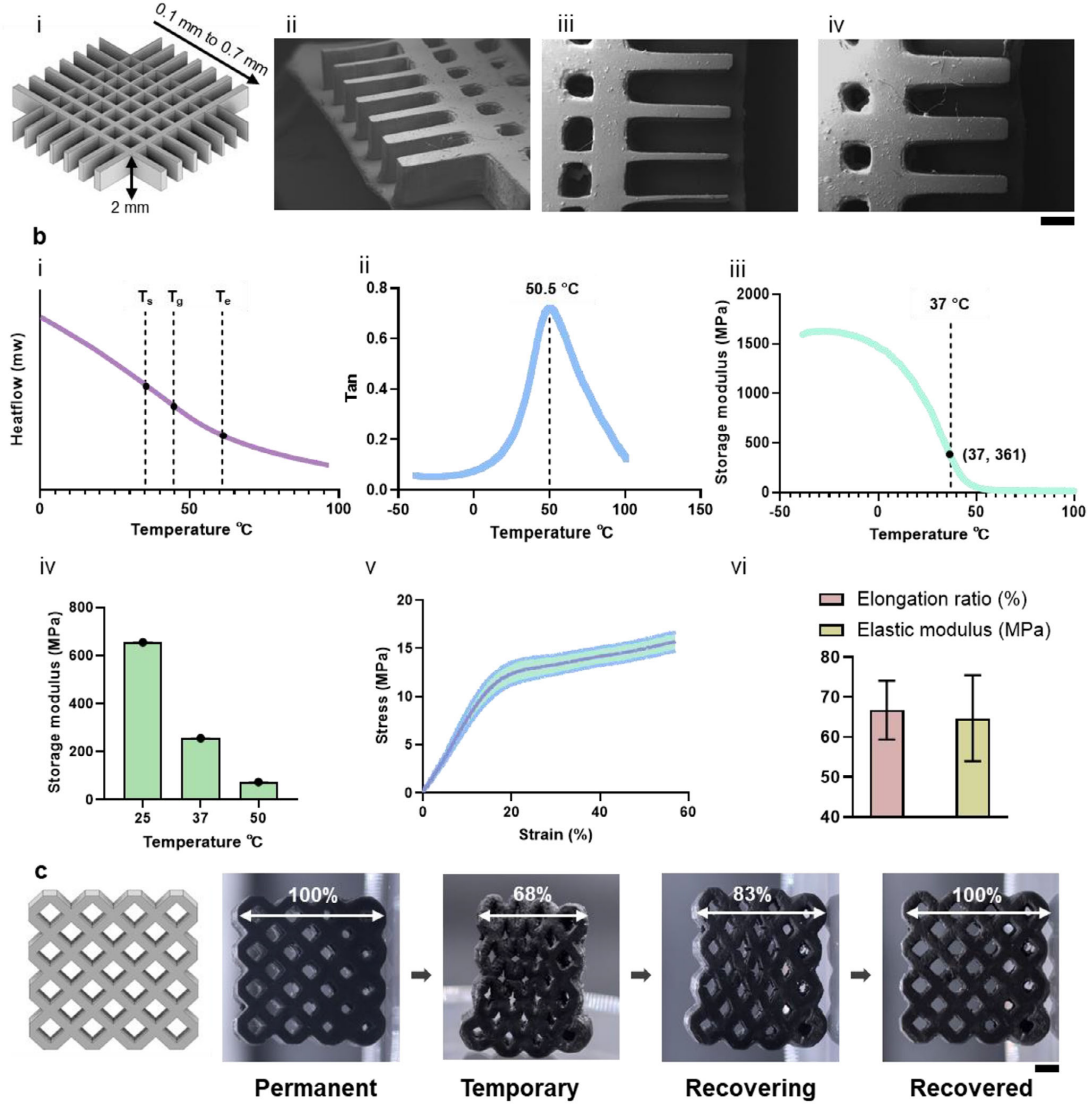
Characterization of the optimal printed material for shape‐memory functionality. Scale bar = 1 mm. (a) (i) 3D model of the mesh structure; (ii–iv) SEM images from different angles showing strip width and height, demonstrating a minimum resolution of 0.1 mm. (b) Thermal and mechanical property characterizations. (i) DSC curves of the optimal resin; (ii, iii) DMA results showing tan δ and storage modulus curves; (iv) Isothermal DMA at three temperatures; (v, vi) Tensile test results at room temperature (*n* = 6). (c) Shape‐memory function validation at room temperature.

The printability of the optimized resin was critical for subsequent stent fabrication and was first evaluated using a mesh structure printing test (Figure 5a). The mesh featured variable strip widths ranging from 0.1 to 0.7 mm and a height of 2 mm. Printing accuracy was assessed via scanning electron microscopy (SEM). SEM images revealed smooth surfaces and well‐defined strips across all widths, demonstrating a lateral resolution of at least 0.1 mm in the XY plane. Mild overcuring was observed in the mesh pores, likely due to residual resin curing during exposure for subsequent layers. This effect can be mitigated by slightly adjusting exposure times, which may require case‐by‐case optimization for specific geometries. Overall, the test confirmed excellent printability and the capability of the resin to produce complex structures with high precision.

Since DSENO deployment relies on its shape‐memory function, the thermal–mechanical behavior of the optimized resin was characterized via dynamic mechanical analysis (DMA). Compared with the broad glass transition curves observed by DSC (Figure 5b‐i), DMA provided a sharper *T*_g_ transition and revealed the storage modulus variation with temperature. The *T*_g_ was ≈50°C, consistent with DSC results (Figure 5b‐ii), and the storage modulus decreased sharply upon heating from more than 1500 MPa to 70 MPa (Figure 5b‐iii), indicating good potential for structural compression. For stenting, however, the material must remain sufficiently stiff post‐deployment to scaffold the vessel wall [62]. Isothermal DMA tests at 25°C, 37°C, and 50°C showed storage moduli of 255 MPa at 37°C and 73 MPa at 50°C (Figure 5b‐iv)—both exceeding the reported modulus of native arteries [63, 64, 65]. These values suggest that during thermal activation, the stent will be stiff enough to expand against vessel contraction. In addition to stiffness, DSENO's shape‐morphing function requires sufficient elasticity to allow significant distortion without fracture. Tensile tests (ASTM D638 type I, room temperature) at room temperature showed a yield strain of ≈18% (Figure 5b‐v) and an elongation at break of ≈65% (Figure 5b‐vi). Together with DSC and DMA findings, these results confirm the material's capability for substantial shape deformation at ≈50°C without mechanical failure.

**FIGURE 6:**
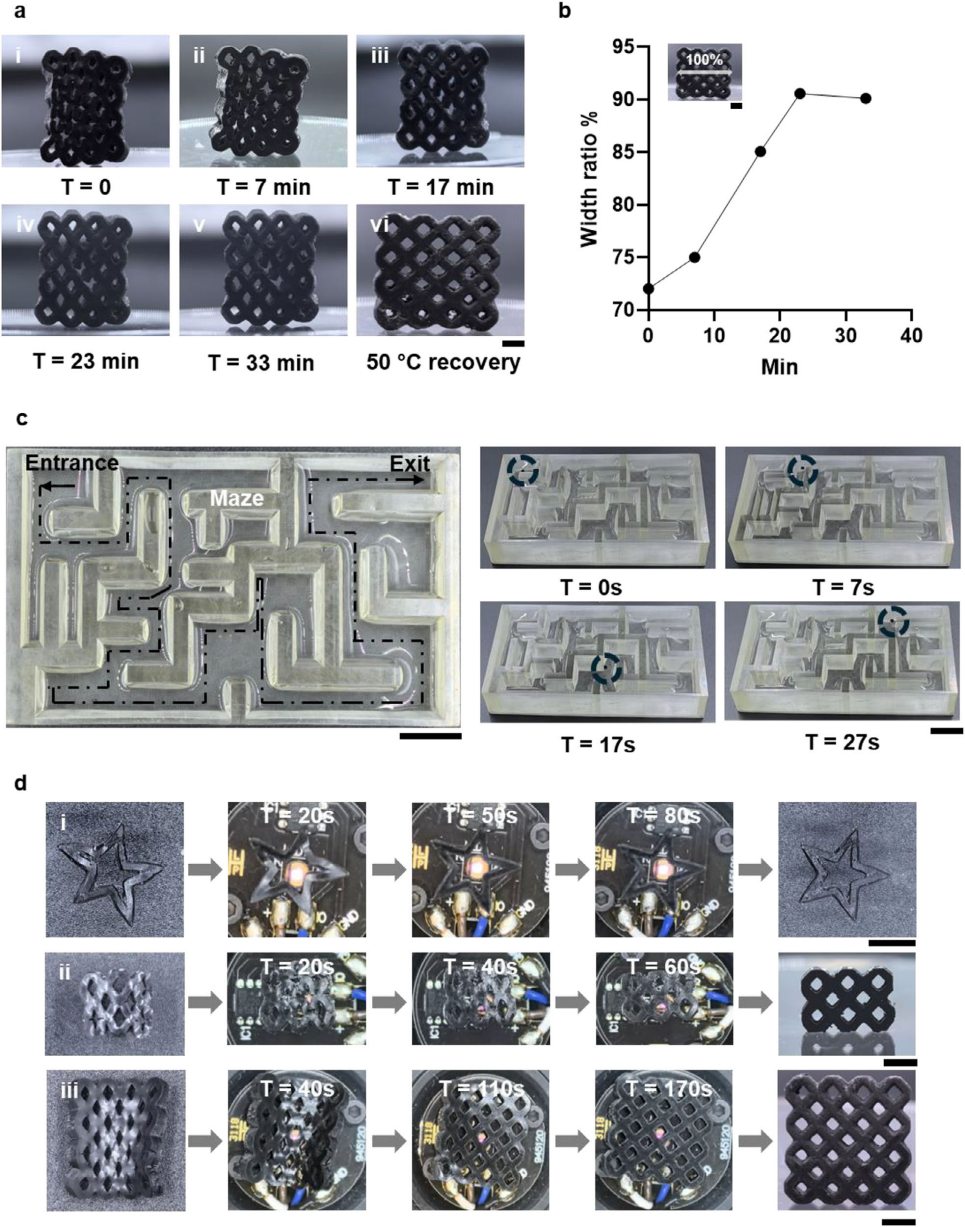
Incubation at 37°C and untethered control investigations. (a) 37°C incubation test: (i) compressed structure; (ii–v) gradual deployment over time; (vi) rapid recovery upon heating to 50°C. Scale bar = 1 mm. (b) Width changes measured during the 37°C incubation test. (c) Magnetic manipulation of a cylindrical structure navigating through a maze. Scale bar = 1.5 cm. (d) Photothermal activation demonstrated on three different compressed structures. Scale bar = 2 mm.

To validate the shape memory polymer function directly, we 3D printed a porous structure. This structure was compressed at 50°C and then cooled to room temperature, successfully fixing it into a temporary shape. This process demonstrates the material's ability to be mechanically deformed and then locked into a new configuration, a key characteristic of shape‐memory polymers (Figure 5c). The memory of the permanent shape was presented when the deformed structure was heated back to 50°C for relaxation. With the demonstration of temporary shape locking and permanent shape recovering, the shape‐memory function was sufficiently demonstrated. Given the physiological working environment, the operational window for precise mobility was investigated by evaluating the self‐deployment of the stent due to the *T*_s_ lower than 37°C. With the same porous structure, the compressed sample was incubated at 37°C, showing gradual recovery (Figure 6a). Significant recovery occurred after 23 min (Figure 6b), while minimal deployment was observed within the first 7 min. After 33 min, heating to 50°C accelerated the process to full recovery. These results suggest an operational window of approximately 10–15 min, during which DESNO could be navigated to the treatment site before substantial deployment began. This operational window was closely linked to untethered control, which warrants further investigation.

### 2.4 Proof‐of‐Concept of Untethered Control for the Material

Magnetic control has been widely exploited in biomedical applications to improve the delivery of drugs by using iron oxide nanoparticles [66, 67, 68], and was therefore incorporated into DSENO design to enable stent navigation within blood vessels. To demonstrate this capability, a maze (Figure 6c) was fabricated and filled with deionized water (DI water). A cylindrical structure (2 mm diameter, 2 mm height), printed with the DSENO material, served as the test object. Guided by an external magnet, the cylinder moved rapidly through the maze following the magnet's trajectory (Figure 6c; Video S1). The cylinder completed the maze in 30 s, demonstrating excellent magnetic responsiveness and the potential for precise, agile control of DSENO in vivo. Another critical control aspect is achieving rapid in situ deployment upon arrival. Leveraging the photothermal effect of embedded Fe_3_O_4_ nanoparticles [69, 70], remote heating via near‐infrared (NIR) light was explored as a trigger mechanism. Given its superior tissue penetration, an 850 nm NIR light source was employed to activate the photothermal response and induce shape recovery. The applied power density of the NIR light source was approximately 26 mW cm^−^^2^ (Figure S21), which falls within the range of low‐level LED therapies (10–200 mW cm^−^^2^) [71, 72, 73, 74], indicating a favorable safety profile for potential clinical use.

To evaluate efficacy, three distorted printed structures were irradiated with NIR light to observe shape recovery (Figure 6d). The star‐shaped structure fully recovered its original form within 80 s, despite most light passing through its void spaces (Video S2). The 6‐repeat‐unit porous structure recovered even faster, within 60 s (Video S3), likely due to more direct light exposure and improved heating efficiency. Photothermal activation efficacy and deployment time were influenced by the direction of light exposure and the size of the structure. The larger 16‐repeat‐unit structure required approximately 170 s longer for full deployment (Video S4), but still completed recovery within 3 min. To confirm the photothermal effect, the star and 16‐repeat‐unit structures were retested, consistently showing rapid NIR‐triggered deployment (Figure S24).

The untethered control of material movement and deployment further exhibited the feasibility of fabricating DSENO using this material. These intrinsic material properties are essential for realizing the full functional potential of DSENO. In addition to material characteristics, the fabrication method and DSENO architecture are equally critical for harnessing these properties effectively, ultimately ensuring the functionality and applicability of DSENO in future vascular interventions.

### 2.5 DSENO Design Diversity and Optimization

The size of vascular stents varies depending on artery location and individual patient differences, necessitating diverse stent designs to accommodate various anatomical requirements [75, 76, 77]. Advances in personalized and precision medicine are driving a revolution in fabrication methods, with 3D printing emerging as a leading solution due to its design flexibility and ease of production [78]. Continuous improvements in 3D printing technology have enhanced printing quality, scalability, productivity, and cost‐effectiveness [79, 80, 81]. Given the need for case‐specific stent designs, 3D printing—specifically LCD 3D printing as previously described—was chosen for DSENO fabrication.

**FIGURE 7:**
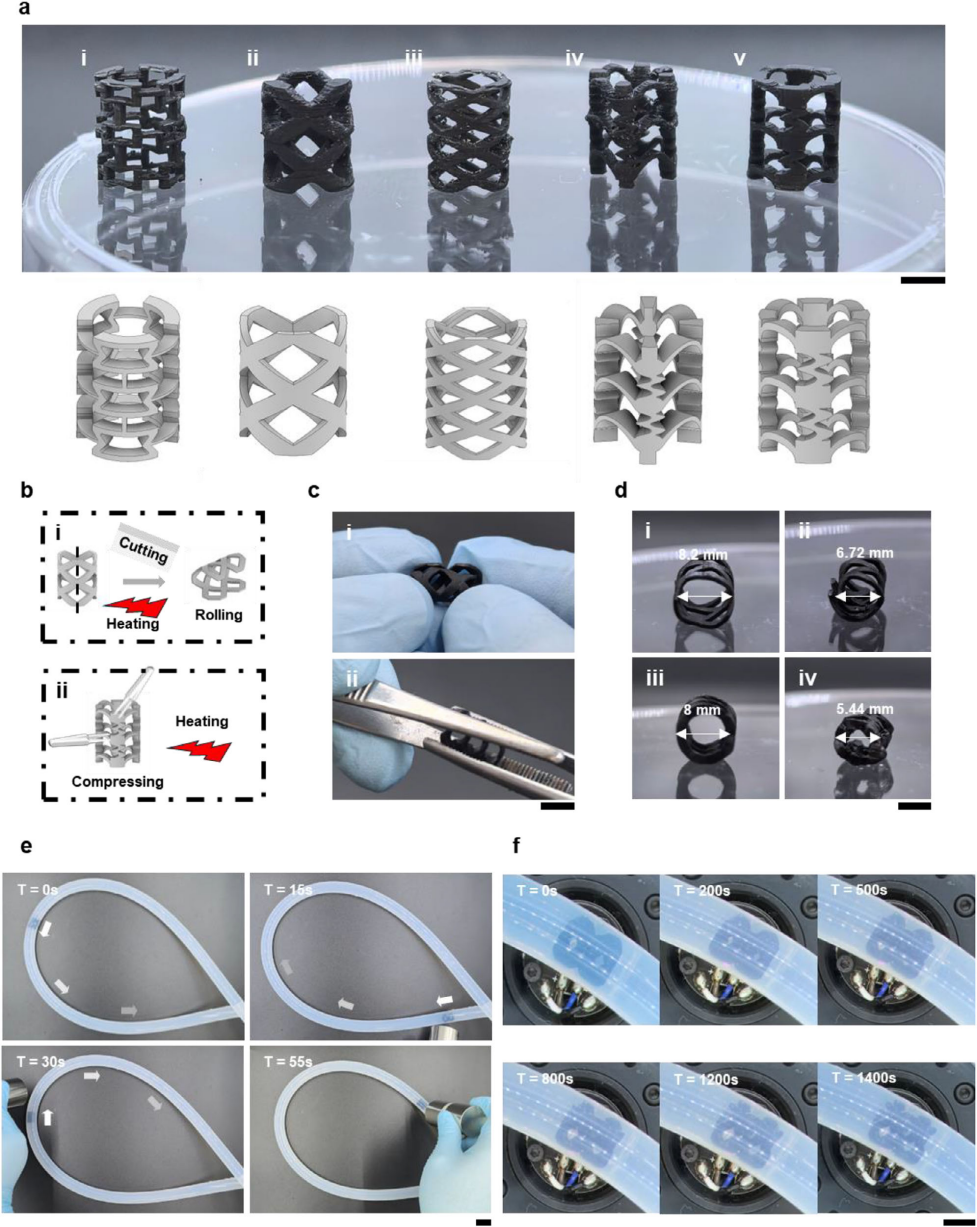
DSENO design optimization and working mechanism demonstration. Scale bar = 5 mm. (a) Five basic DSENO stent designs. (b) Schematics of compression methods: (i) method for type II and III designs; (ii) method for type IV and V designs. (c) Photos of practical compression operations: (i) rolling of a type II design sample; (ii) compression of a type V design sample. (d) Size changes before and after compression: (i, ii) type II design; (iii, iv) type V design. (e) Magnetic control of a type II DSENO stent moving inside a silicone tube. (f) Time‐lapse images showing NIR‐induced photothermal deployment of a type II DSENO stent.

The demonstrated print quality enabled fabrication of complex structures capable of programmable shape‐morphing without disassembly. Five primary DSENO designs (Figure 7a) and their variants (Figures S26–S30) were printed to evaluate shape‐morphing performance. The first design employed a 3D auxetic structure (Type I) intended to shrink in both height and width simultaneously. While height shrinkage was significant, width shrinkage was negligible, likely due to overcuring at structural corners below the printer's resolution, which eliminated space needed for lateral shrinkage (Figure S26). Additionally, the hollow centre reduced the theoretical shrinkage ratio compared to reported auxetic structures. These challenges led to replacing the first design with alternative concepts.

Types II and III were based on traditional stents but differed in repeat unit count: Type II featured fewer, larger pores; Type III had more, smaller pores. Traditional stents are typically thin and long cylinders that expand in diameter but shorten in length upon balloon inflation. Although Types II and III could be fixed in a compressed, long, and thin form, their increased length reduced mobility during transport. To address this, Types II and III employed a cutting and rolling compression method (Figure 7b‐i), decreasing diameter without increasing length. The original Type II DSENO (1.5 mm thick) was difficult to roll, so the thickness was reduced to 0.6 mm to facilitate rolling after heating (Figure 7c‐i). While cutting could risk active breakage affecting deployment, recovery tests showed that the flattened, cut Type II stent fully recovered its cylindrical shape upon NIR activation (Figure S27 and Video S5). The diameter decreased from 8.2 to 6.72 mm post‐compression (Figure 7d‐i,ii). Type III, having thinner strips, required more delicate rolling and exhibited a smaller diameter reduction (Figure S28).

Types IV and V featured bulky frames connected by curved bridges designed to bend upon heating. These designs were compressed orthogonally using tweezers during heating (Figure 7b‐ii). Type IV's diameter decreased by 28% (Figure S29). A variant with three repeat units wrapped instead of four compressed but showed less shrinkage, possibly due to reduced contact area with tweezers. Compared to Type IV, Type V optimized bridge connections by thickening them and linking to a larger rectangular frame, enabling a more substantial diameter reduction from 8 mm to 5.44 mm (Figure 7d‐iii,iv). In summary, Type II demonstrated superior performance using the cutting and rolling method, while Type V excelled with orthogonal compression. The diversity of compression methods and shrinkage ratios summarized in Table 1, stemming from DSENO's varied designs, highlights the flexibility and convenience of 3D printing fabrication. This versatility could enable personalized stent designs tailored for improved clinical outcomes.

**TABLE 1:** Size changes of different DSENO designs after compression.

Design Number	3D Model	Compression Method	Height Decrease Ratio	Diameter Decrease Ratio
i	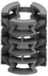	Compressing from the top	17%	0.8%
ii	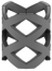	Cutting and rolling	0%	18%
iii	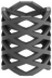	Cutting and rolling	0%	11%
iv	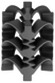	Compressing from the sides	0%	28%
v	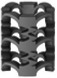	Compressing from the sides	0%	32%

### 2.6 DSENO Functionality Demonstration

The proposed advantages of DSENO for untethered spatiotemporal control of deployment and stenting were validated using an in vitro tube model. A compressed Type II DSENO stent was inserted into a silicone tube with an inner diameter of 8 mm (Figure S31a). The tube was fully filled with DI water to simulate blood vessels. Because the compressed stent was smaller than the tube diameter and immersed in water, friction between the stent and tube was minimal, facilitating stent mobility. Under these conditions, a magnet was employed to navigate the stent smoothly through the tube (Figure 7e; Video S6), demonstrating effective magnetic control for movement within vessel‐like environments.

Deployment within the tube was also tested by illuminating the DSENO stent with NIR light (Figure 7f; Video S7). In the aqueous environment, the photothermal effect was less pronounced due to heat dissipation into the surrounding water. Nonetheless, noticeable shape recovery began after 1 min and was completed gradually within 30 min. To assess stenting functionality, the magnet was used to attempt movement of the stent after deployment; the stent remained firmly in place without shifting (Figure S31b and Video S7), indicating successful expansion and vessel wall engagement. Further validation involved sectioning the tube to observe the stent cross‐section (Figure S31c). The results confirmed sufficient DSENO expansion and attachment to the inner tube wall. The hollow centre diameter was measured at 6.8 mm (Figure S31c‐iii), consistent with effective deployment.

**FIGURE 8:**
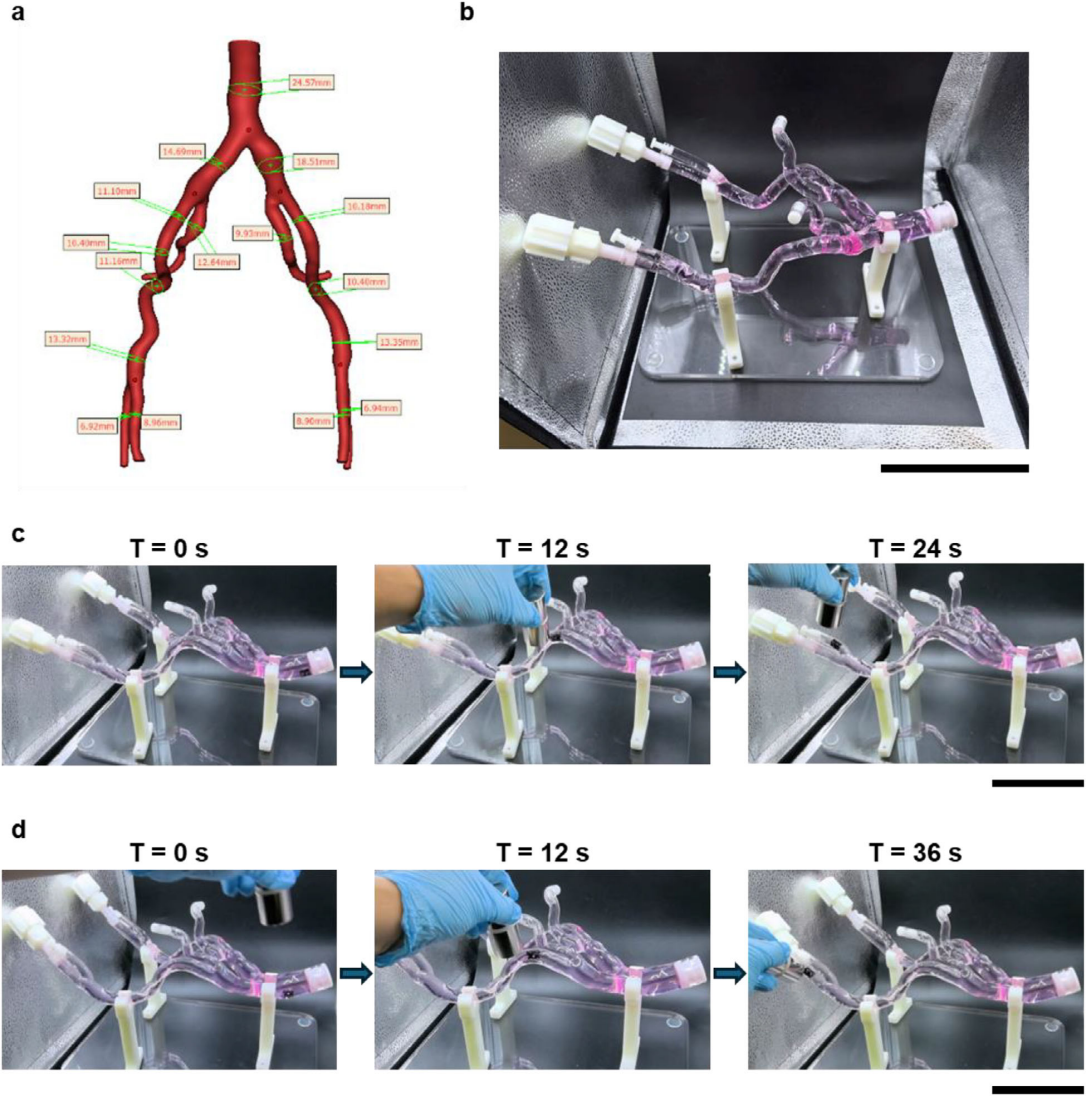
PDMS fabricated vessel model for DESNO magnetic control validation. Scale bar = 15 cm. (a) Schematic figure of the vessel model with inner diameter labelled. (b) The practical vessel model is infilled with microvascular endothelial cell growth medium. (c) Magnetic control of a type II DSENO stent moving to one end of the model. (d) Magnetic control of a type II DSENO stent moving to another end of the model.

To further investigate the magnetic navigation capability of DESNO, an advanced PDMS vessel model (Figure 8a,b) was employed to evaluate stent movement in a more complex environment. The vessel model was fully filled with cell culture medium to mimic physiological fluid conditions, and a compacted type II DESNO stent was introduced into the model. Compared with the straight tube tests, this vessel model incorporated increased complexity, including multiple curvatures, bifurcations, changes in elevation, and variations in inner diameter, thereby providing a more stringent assessment of magnetic navigation performance. During the experiments, the stent was successfully guided by an external magnet from the broader inlet to the narrower outlet, including navigation through curved and branched regions (Figure 8c,d; Video S10). These results demonstrate that DESNO can be reliably magnetically guided in a vessel‐mimicking environment with complex geometries. Collectively, these findings confirm that DESNO achieves controlled navigation and effective stent deployment under conditions relevant to physiological vascular architectures.

## 3 Conclusion

We have fabricated the DSENO stent system, designed to achieve magnetically controlled navigation to the treatment site, where it can deploy and scaffold narrowed vessels as a permanent implant with remote spatiotemporal control. To validate the concept and feasibility, the printing material was optimized based on thermal and mechanical properties, surface modification efficiency, NO catalytic capability, and biocompatibility. These thermal and mechanical properties enabled DSENO to undergo shape‐morphing from a compressed to an expanded state, fulfilling its stenting function with precise control. Promising NO release and biocompatibility data demonstrated that surface modification effectively enhances endogenous NO production, potentially alleviating acute inflammation and resisting late‐stage restenosis. Leveraging the high printability of the optimized material, five DSENO designs and their variants were evaluated for compression ratios, highlighting the advantages of 3D printing in design flexibility and fabrication convenience. Finally, an in vitro tube model confirmed DSENO's robust magnetic mobility and spatiotemporally controlled deployment, underscoring its promise for future clinical translation.

Addressing key challenges in current angioplasty and stenting treatments, DSENO offers a promising alternative to drug‐eluting stents and bioresorbable stents with several advantages: (i) By replacing catheter and balloon assistance, DSENO enables remote‐controlled transportation and deployment, reducing tissue damage and lowering the risk of acute restenosis; (ii) Endogenous NO catalysis via surface modification potentially overcomes the limitations of drug consumption, allowing DSENO to serve as a permanent implant without mechanical failure while resisting late‐stage restenosis; (iii) The diversity in DSENO designs and compression methods provides tailored options to meet individual patient needs, aligning with the emerging trend toward personalized and precision medicine.

While the current study demonstrates the mechanical, catalytic, and functional efficacy of the DSENO stent platform under physiologically relevant in vitro and ex vivo conditions, we acknowledge that in vivo evaluation in animal models remains a critical future milestone. Complex systemic factors—such as the foreign body response, long‐term hemodynamic remodeling, and chronic inflammation—can only be fully captured in live vascular systems. Our ongoing efforts are focused on refining the stent geometry for porcine model implantation and establishing a multi‐month follow‐up protocol to evaluate chronic restenosis prevention. The results presented here serve as a necessary regulatory and scientific baseline for these upcoming preclinical investigations.

## 4 Experimental Section

### 4.1 Materials

All chemicals and reagents were directly used without any further processing. 1,3,5‐triallyl‐1,3,5‐triazine‐2,4,6(1H,3H,5H)‐trione (TATATO, Sigma‐Aldrich 114235), tris(3‐mercaptopropionate) (TMTMP, Sigma‐Aldrich 381489), tricyclon[5.2.1.02,6], decanedimethanol diacrylate, (TCMDA, Sigma–Aldrich 496669), 2,5‐bis(5‐tert‐butyl‐benzoxazol‐2‐yl)thiophene, (CB+, Sigma‐Aldrich 223999), 2‐benzyl‐2‐(dimethylamino)‐ 4’‐morpholinobutyrophenone (I‐369, Sigma‐Aldrich 405647), and iron (II, III) oxide (Sigma–Aldrich 637106) were used to make printing resin candidates of DSENO. Ethanol (Merck, 1.00983.2511) and acetone (Sigma–Aldrich, 904082) were used for cleaning. Phosphate‐buffered saline tablets (OXOID, X6571f) were used to make a 1× PBS solution for incubation tests. Standard clear resin (Anycubic) was used to print the maze. Biology reagents DPBS (14190‐144), alamarBlue cell viability reagent (DAL1025), Hoechst 33342 (62249), and DAF‐FM diacetate (D23842) were purchased from Thermo Fisher. LIVE/DEAD viability/cytotoxicity kit for mammalian cells was purchased from Invitrogen. GSNO was purchased from MedChemExpress.

### 4.2 Printing Resin Preparation and Fabrication

3D Printing and Resin Formulation: 3D models were designed using Autodesk Inventor and exported as STL files. Printing resins were prepared by mixing TATATO, TMTMP, TCMDA, CB+, and I‐369 according to the ratios in Table S1, followed by the addition of 2 wt.% Fe3O4 nanoparticles.

Fabrication was performed using an Anycubic Mono SE LCD 3D printer (light intensity: 2.5 mW/cm^2^). The printing parameters were optimized to account for the light‐shielding effect of the magnetic nanoparticles:

1. Initial Layers (×5): 90 s exposure (to ensure strong plate adhesion).
2. Subsequent Layers: 60 s exposure.
3. Layer Thickness: 50 µm.
4. Lift Speed: 2 mm/s (optimized to minimize peel‐force‐induced delamination).

Engineering Optimization: A vertical printing orientation was adopted to circumvent the need for internal support structures within the stent's lumen. By integrating a flat, sacrificial base (raft) at the bottom of the stent, we ensured stable adhesion to the build plate while maintaining the circularity and surface integrity of the tubular geometry. Post‐printing, samples were washed in isopropyl alcohol and post‐cured for 5 min to ensure maximum monomer conversion.

### 4.3 Ellman's Assay for Thiol Quantification

A 0.1 M sodium phosphate buffer (pH 8.0) containing 1 mM EDTA was prepared as the reaction medium. DTNB solutions (2 and 1 mg/mL) were prepared in this buffer. L‐cysteine was used as the reference standard, and serial dilutions were prepared to generate concentrations ranging from 10 to 0.039 mM. The 3‐arm thiol monomer was initially considered as a control but was excluded due to poor solubility in aqueous buffer. For the assay, L‐cysteine solutions were mixed with DTNB (2 mg/mL) at a 1:1 volume ratio (final reaction volume = 400 µL) and incubated at room temperature for 10 min with gentle shaking. The absorbance of the supernatant at 412 nm was measured using a microplate reader (100 µL per well, triplicate for each condition). A 1 mg/mL DTNB solution was used as the blank to subtract background signals.

### 4.4 Printed Plate Coating with PEI

Material candidates were printed in small square plates with a 5 mm length and 1 mm thickness. Plates were divided into two groups and pre‐cleaned by sequential sonication in ethanol (1 min × 5) and PBS (1 min × 3). Polyethylenimine (PEI, 10 mg/mL, 0.4 mM in PBS) was prepared and mixed with crosslinkers at different molar ratios to primary amines (2.5%, 5%, 10%, and 25%). The pH was adjusted to 7.4 to favor both NHS ester and Michael addition reactions. Group 1 was treated with NAS, and Group 2 with MAS. Solutions were stirred from 0°C to room temperature for ≈2 h to allow pre‐conjugation of crosslinkers to PEI. The resulting PEI–crosslinker solutions were incubated with plates for 72 h, followed by sonication washing in PBS (1 min × 5). Plates were dried with nitrogen gas before further use.

### 4.5 Quantification of Cumulative No Release by Griess Assay

Nitric oxide release was quantified using the Griess assay. GSNO (50 µm) was used as the NO donor, and plates subjected to a clean procedure only were used as blank controls. Uncoated and coated samples were incubated in HEPES buffer (pH 7.4) supplemented with 1 mM EDTA at 37°C for 24 h. After incubation, aliquots of the supernatant were collected and reacted with the Griess reagent according to the manufacturer's instructions. The absorbance was measured at 540 nm using a microplate reader, and nitrite concentrations were determined by comparison with a sodium nitrite standard curve.

### 4.6 Material Thermal and Mechanical Properties Characterizations

Differential Scanning Calorimetry (DSC) was employed to measure the glass transition temperature (T_g_). Samples were first heated from room temperature to 100°C, then cooled to 0°C, followed by a second heating to 100°C. The second heating curve was used for analysis. Measurements were conducted on a TA Q20 DSC with a heating/cooling rate of 10 K/min.

Dynamic Mechanical Analysis (DMA) was performed using a TA Q800 with a liquid nitrogen cooling accessory. Samples measuring 40 mm × 8 mm × 0.8 mm were tested via single cantilever bending. Temperature ramp tests were conducted from −40°C to 100°C at 3 K/min with a bending frequency of 1 Hz and a displacement amplitude of 15 µm. Isothermal tests were conducted at fixed temperatures of 25°C, 37°C, and 50°C. To evaluate the long‐term mechanical durability of the 3D‐printed DSENO materials under physiological conditions, extended cyclic DMA testing was performed at 37°C. As illustrated in Figure S20, the storage modulus (E') and tan delta remained remarkably stable throughout the 140‐h duration at a frequency of 2 Hz, representing over 106 loading cycles. No significant mechanical degradation, micro‐cracking, or accumulation of plastic deformation was observed, confirming the robust structural integrity of the crosslinked polymer network. This sustained mechanical stability is critical for vascular applications, where the stent must withstand continuous hemodynamic cyclic loading without compromising its radial support or surface functionality. The absence of significant creep or fatigue‐induced softening during this ultra‐extended evaluation further underscores the suitability of the DSENO candidates for long‐term implementation as permanent vascular implants.

Tensile tests were performed on a Mark–10 ESM303 tensile tester equipped with a 1 KN force gauge (model M5–5). The displacement speed was set at 1.1 mm/min. Samples were prepared following the ASTM D638 Type I standard.

### 4.7 Scanning Electron Microscope (SEM) and Energy Dispersive X‐Ray Spectroscopy (EDS)

SEM was used to validate print quality and assess material stability. Samples were mounted on conductive tape and sputter‐coated with a 15 nm platinum layer using a Quorum Q300T Plus coater. Imaging was performed on a Zeiss Auriga SEM at 5 kV accelerating voltage with a 30 µm aperture. EDS elemental mapping was performed with the same SEM equipped with an Oxford Instruments EDS detector, operated at 20 kV and a 120 µm aperture under high voltage mode.

### 4.8 Material Stability and Degradation Tests

Material candidates A and B (Table S2) were printed into discs (5 mm diameter, 0.5 mm thickness) and incubated in 1× PBS at 37°C for 30 days. Samples were collected at days 1, 3, 5, 10, 20, and 30, with PBS refreshed at each time point. After collection, samples were rinsed with DI water and gently dried with paper towels. Wet weights were recorded immediately. Samples were then freeze‐dried overnight for dry weight measurement and SEM imaging.

### 4.9 Long‐Term Material Stability and Degradation Tests in PBS and EGM‐2 MV

Uncoated and coated materials A and C were immersed in 1× PBS and EGM‐2 MV at 37°C for 60 days. The media was refreshed every 3 days. After incubation, all samples were air‐dried and sputter‐coated with platinum for SEM imaging without additional treatment to preserve the original surface state.

### 4.10 Shape‐Memory Function and Photo‐Thermal Effect Validation

Printed samples were heated to 50°C in an oven and compressed to a temporary shape. The compressed samples were rapidly cooled in room‐temperature DI water to fix the shape. For recovery, samples were reheated to 50°C to return to their permanent shape. Photothermal effects were tested by exposing compressed samples to 850 nm LED light (Thorlabs M850LP1) controlled by a Thorlabs LED driver (LEDD1B).

### 4.11 Vessel Model Test

A PDMS vessel model (Zhiling, China) was fully filled with microvascular endothelial cell growth medium (EGM‐2 MV). A compacted Type II DSENO stent was inserted into the model from the wider end as the starting point. An external magnet was then used to guide the stent through the vessel model toward the narrower end.

Atomic force microscopy (AFM): All AFM measurements were performed on a Bruker Dimension ICON SPM equipped with a Nanoscope V controller (software version 9.70). Tapping mode imaging was carried out using an OTESPA‐R3 probe (Bruker). Scans were conducted at sizes of 1 µm and 300 nm, with a scan rate of 0.6–0.7 Hz and a peak force of approximately 500 pN. The feedback gain was adjusted to optimize surface tracking while minimizing noise. Image resolution was set to 512 pixels/line for the 1 µm scans and 256 pixels/line for the 300 nm scans. The arithmetic average roughness (Ra) and root mean square roughness (Rq) were calculated using NanoScope Analysis software (Bruker) according to standard definitions:

$$Ra=\frac{1}{n}\;\sum_{i=1}^{n}\left|Zi\right|$$

$$Rq=\sqrt{\frac{1}{n}\sum_{i=1}^{n}Zi^{2}}$$

NIR LED light intensity measurement: the irradiance of the 850 nm LED (Thorlabs M850LP1), controlled by a Thorlabs LED driver (LEDD1B), was measured using an infrared irradiance meter (LH‐129, Puyan). The measurements were repeated three times, and for each measurement, the irradiance was recorded twice.

### 4.12 Cyclic NIR Tests

A flat printed sheet (6 mm × 8 mm × 0.5 mm) was irradiated using an 850 nm LED (Thorlabs M850LP1) controlled by a Thorlabs LED driver (LEDD1B). The temperature variation was monitored in real time using an infrared thermal imaging industrial thermometer (E09, HIKMICRO).

### 4.13 Fourier‐Transform Infrared Spectroscopy (FTIR)

FTIR was conducted on a Bruker Alpha FTIR spectrometer equipped with room temperature DTGS detectors. After background calibration, the PEI‐treated materials were placed on the crystal plate for testing. An absorption spectrum was then obtained by scanning the material from 400–4000 cm^−1^.

### 4.14 X‐ray Photoelectron Spectroscopy (XPS)

XPS was performed on an ESCALAB 250Xi (Thermo Scientific) with the binding energies calibrated to the C 1s line at 284.8 eV.

### 4.15 Contact Angle

An optical contact angle and surface tension meter (KSV CAM 200 Optical Contact Angle Meter) was utilized to measure the wettability of the printed sample plates. 2 µL ultrapure water droplets were deposited onto the substrate surfaces, and images were taken immediately after droplet placement.

### 4.16 Cell Culture and Biocompatibility

The biocompatibility of material candidates was investigated using human umbilical vein endothelial cells (HUVECs) from Lonza Bioscience below passage 12. HUVECs were cultured in Microvascular endothelial cell growth medium (EGM‐2 MV) and sub‐cultured at 80–90% confluency. Human coronary artery smooth muscle cells (HCASMCs) from Cell Applications were maintained in human smooth muscle cell growth medium (311–500) and passaged at 70–90% confluency. Cells were seeded in 24 well plate at a density of 2 × 10^4^ cells/500 µL/well and stabilized for 24 h at 37°C in a 5% CO_2_ incubator. Both PEI‐coated and uncoated plates of material candidates were sterilized and rinsed with DPBS 3 times prior to cell exposure. Cell viability was evaluated using both transwell‐based indirect contact and direct cell–material contact configurations, with materials placed either in 3D‐printed PLA transwell inserts or directly in contact with cells, respectively. After 48 h incubation, the transwells or material samples were removed, and cell viability was assessed through the alamarBlue assay

### 4.17 Extract Preparation and Cell Viability with the Extract

To evaluate the potential toxicity of leachable components, extractable cytotoxicity assays were performed in accordance with ISO 10993–5. DSENO material samples were incubated in serum‐supplemented microvascular endothelial cell growth medium (EGM‐2 MV) at 37°C. Specifically, two 3D‐printed specimens were placed in each well of a 24‐well plate containing the culture medium. Following the extraction period, the conditioned media (extracts) were collected and diluted with fresh EGM‐2 MV at a 1:1 (v/v) ratio to prepare the test medium.

### 4.18 Cell Culture and Treatment

HUVECs were seeded into 24‐well plates at a density of 2 × 10^4^ cells per well in 500 µL medium and allowed to stabilize for 24 h before the cytotoxicity test, and allowed to adhere under standard physiological conditions (37°C, 5% CO2). The culture medium was then replaced with the prepared extract‐based test medium, and the cells were incubated for an additional 48 h.

Cell Viability Quantification: Following incubation, cell viability was quantitatively assessed using the Alamar Blue assay. The fluorescence/absorbance was measured to evaluate metabolic activity. Cell viability was calculated as a percentage relative to the blank control (cells cultured in fresh, non‐conditioned medium):

$$\mathrm{Cell}\;\mathrm{Viability}\left(\%\right)=A_{\mathrm{sample}}/A_{\mathrm{control}}\times 100\%$$

### 4.19 Confocal Microscopy and Fluorescence Imaging

To evaluate the intracellular NO generation without additional NO donors, samples were stained with DAF‐FM diacetate (5 µM) and nuclear counterstain Hoechst 33342 (1:500) for 30 min at 37°C in a 5% CO2 incubator after 48 h incubation with material candidates. The cells were rinsed with DPBS prior to confocal microscopy imaging using Zeiss LSM 800.

For endothelial function, after incubation with blank control, uncoated, or coated materials, immunofluorescence staining was performed on fixed samples. Cells were fixed with 4% paraformaldehyde for 2 h, followed by permeabilization with 0.2% Triton X‐100 for 30 min, and blocked with 2.5% bovine serum albumin for 30 min prior to immunostaining. Cells were then incubated with primary antibodies for vascular endothelial cadherin (ab33168, Abcam, 1:300) for 2 h, followed by incubation with secondary antibodies, goat anti‐rabbit Alexa Fluor 647 (SAB4600184, Merck, 1:400), Rhodamine Phalloidin (R415, Thermo Fisher, 1:200), and nuclear stain Hoechst 33342 (1:500) for 2 h. Cells were rinsed three times with PBS between each step, and imaging was performed on a Zeiss LSM 800. All fluorescence imaging data were processed using Imaris software (Oxford Instruments), mean fluorescence intensity was measured per cell.

Cell viability and endogenous NO generation data were presented as mean ± standard deviation, with ≥3 independent replicates. Statistical analyses were performed in GraphPad Prism using one‐way ANOVA, statistical difference is denoted as ns = no significance, ^*^p < 0.05, ^**^p < 0.01, ^***^p < 0.001, ^****^p < 0.0001.

### 4.20 In Vitro Hemolysis Evaluation

A healthy adult was recruited to donate blood samples. The subject provided written informed consent, and the study was approved by the University of Melbourne human research and ethics committee (approval 2025‐33501‐71356‐3). Whole blood was collected with sodium heparin anticoagulant. The blood samples were then centrifuged at 3500 g for 5 min at 37°C to isolate red blood cells (RBCs) from plasma. After centrifugation, the RBCs were washed three times with DPBS. Then, the supernatant was discarded, and DPBS was added to obtain a 2% (v/v) RBC suspension. One uncoated or coated printed plate was immersed in 1.5 mL of 2% RBC suspension and incubated for 2 h at 37°C, with water as a positive control and DPBS as a negative control. After incubation, all samples were centrifuged at 9000 g for 5 min, and 100 µL of the sample supernatant was collected and transferred to a 96‐well plate. The absorbance of the supernatant was measured using a microplate reader at 577 nm.

The percentage hemolysis of RBCs was calculated using the following equation:

Where A0 is the absorbance of blood in DPBS, Aw is the absorbance of blood in water, and A1 is the absorbance of blood‐treated plates, all measured at 577 nm.

### 4.21 Whole Blood Coagulation Assay

Whole blood coagulation tests were conducted to qualitatively evaluate the anticoagulant performance of the sample surfaces. Fresh whole blood containing sodium citrate as anticoagulant was re‐calcified by adding a 10% (v/v) solution of 0.2 m CaCl_2_ and gently mixed to initiate coagulation. Subsequently, 25 µL of the activated blood was immediately deposited onto the surface of each sample and incubated at 37°C for 10 or 15 min. After incubation, the samples were gently rinsed with PBS to remove non‐adherent blood components. The coagulation behavior and clot retention on the sample surfaces were then recorded using a digital camera under visible light. As the printed materials were black in color, which limited visual discrimination of blood clots under visible light, ultraviolet (UV) illumination was additionally employed to enhance contrast and qualitatively visualize blood clot adhesion and distribution on the sample surface.

## Conflicts of Interest

The authors declare no conflict of interest.

## Supporting information




**Supporting File 1**: adma72760‐sup‐0001‐SuppMat.docx.

**Supporting File 2**: adma72760‐sup‐0002‐Video S1.mp4.

**Supporting File 3**: adma72760‐sup‐0003‐Video S2.mp4.

**Supporting File 4**: adma72760‐sup‐0004‐Video S3.mp4.

**Supporting File 5**: adma72760‐sup‐0005‐Video S4.mp4.

**Supporting File 6**: adma72760‐sup‐0006‐Video S5.mp4.

**Supporting File 7**: adma72760‐sup‐0007‐Video S6.mp4.

**Supporting File 8**: adma72760‐sup‐0008‐Video S7.mp4.

**Supporting File 9**: adma72760‐sup‐0009‐Video S8.mp4.

**Supporting File 10**: adma72760‐sup‐0010‐Video S9.mp4.

**Supporting File 11**: adma72760‐sup‐0011‐Video S10.mp4.

## Data Availability

The data that support the findings of this study are available in the supplementary material of this article.
