# Supplementary material for: Deployable 3D‐Printed Vascular Stent with Surface‐Catalysed Endogenous Nitric Oxide Generation
Source: Adv Mater. 2026 Mar 15;38(21):e20199. doi: 10.1002/adma.202520199 (PMC13073119; doi:10.1002/adma.202520199)
Supplement: Supplementary file 1 — Supporting File 1: adma72760‐sup‐0001‐SuppMat.docx. [file ADMA-38-e20199-s010.docx]

**Supporting Information**

**Deployable 3D-Printed Vascular Stent with Surface-Catalysed Endogenous Nitric Oxide Generation**

*Kun Zhou^1^†, Zifei Han^1,2^†, Kang Lin^1,2^, Di Wu^1^, Siti Nur Asyura Adzlan^1,2^, Qingqing Fan^3^, Christina Cortez-Jugo^3^, Rona* *Chandrawati^1,2^*, Cyrille Boyer^1,2^**

^1^School of Chemical Engineering, UNSW Sydney, Sydney, New South Wales 2052, Australia

^2^Australian Centre for Nanomedicine (ACN), UNSW Sydney, Sydney, New South Wales 2052, Australia

^3^Department of Chemical Engineering, The University of Melbourne, Parkville, Victoria 3010, Australia

***Corresponding authors. Email:** **rona.chandrawati@unsw.edu.au; cboyer@unsw.edu.au**

†These authors contributed equally to this work


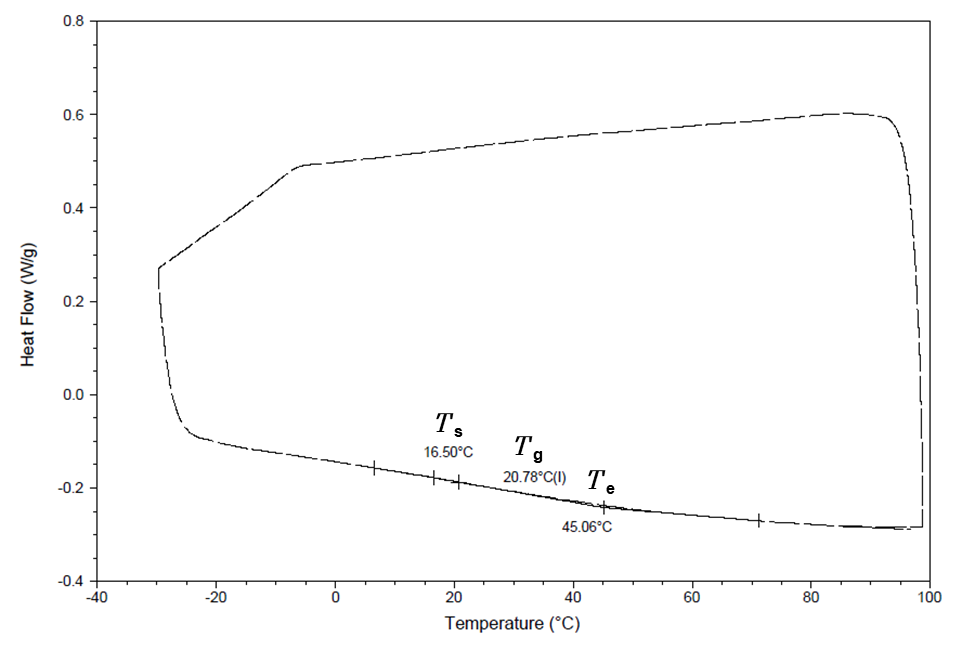


**Figure S1.** DSC curve of resin 1 sample with a heating/cooling rate of 10 K/min exported from the DSC software. The T_s_, T_g_, and T_e_ were calculated based on the secondary heating curve by the software.

**Table S1.** Resin formulation list and crucial temperatures of the printed materials without post-treatment. For Resin 4-5, all samples were post-treated without applicable T_s,_ T_g_ and T_e_ here.


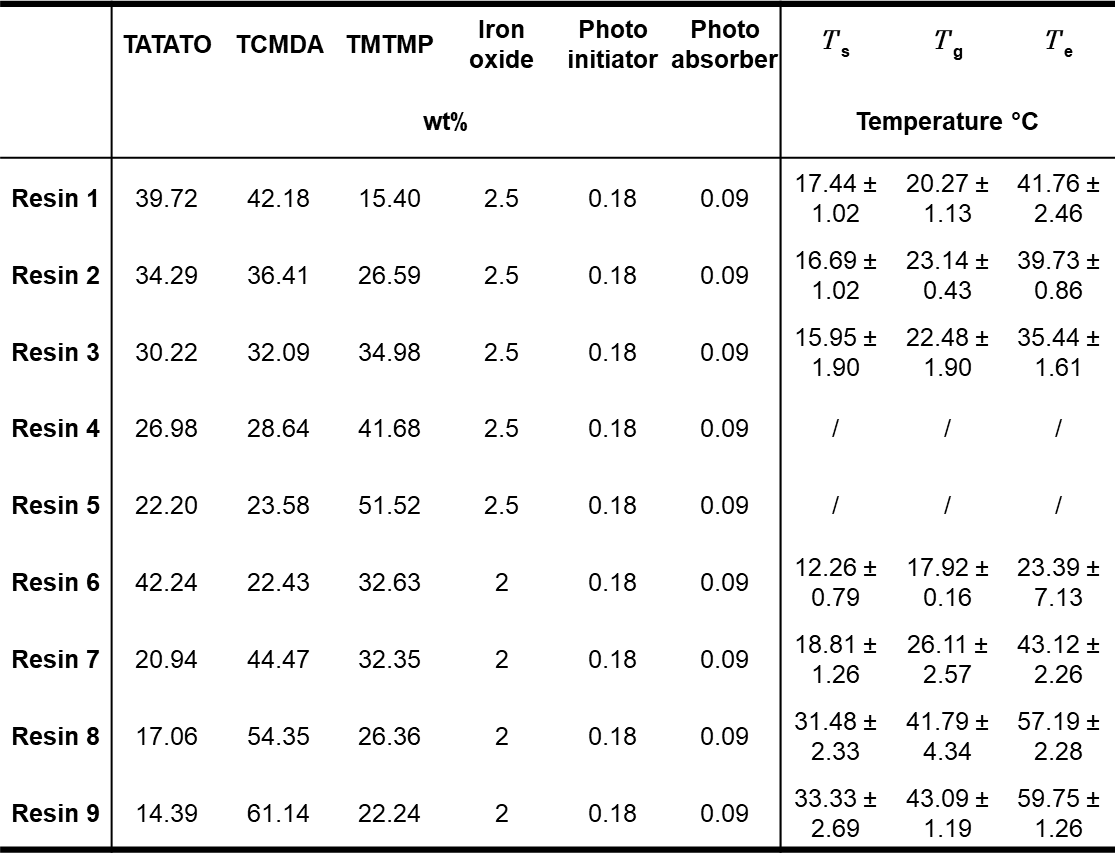


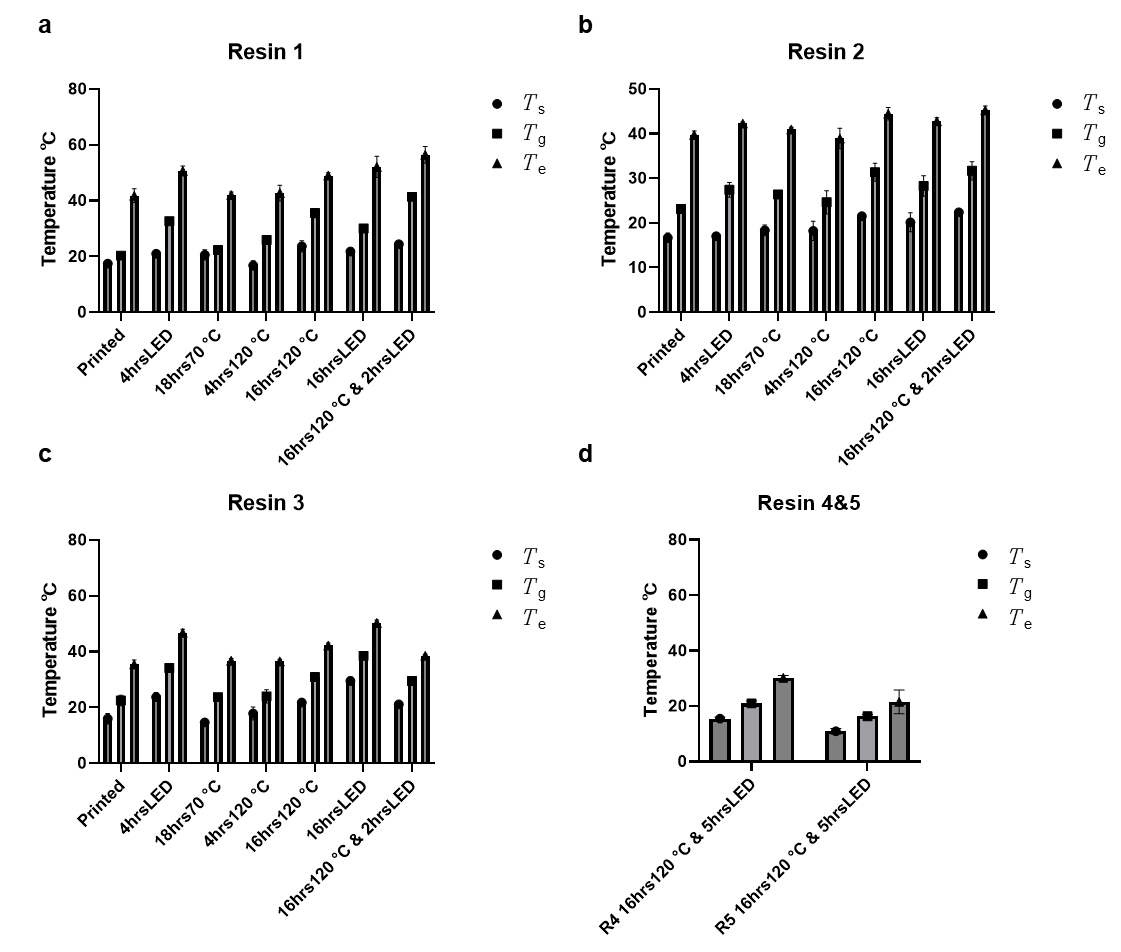


**Figure S2.** The *T*_s_, *T*_g_, and *T*_e_ of printed resin 1-5 with different post-treatments were determined from the second-heating DSC curves. n = 3. (a) Resin 1 and variants. (b) Resin 2 and variants. (c) Resin 3 and variants (d) Variants of resin 4 and resin 5.


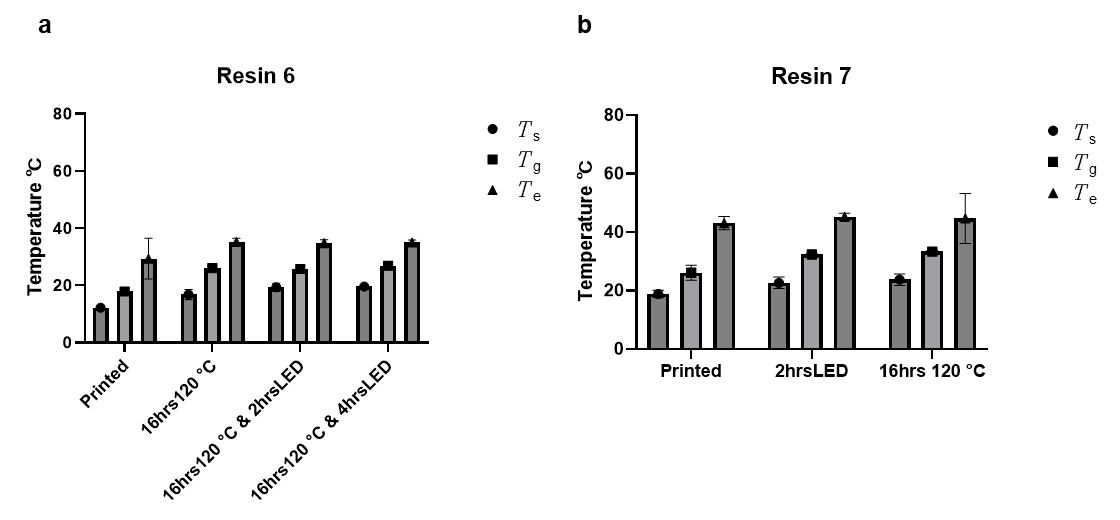


**Figure S3.** The *T*_s_, *T*_g_, and *T*_e_ of printed resin 6-7 with different post-treatments were determined from the second-heating DSC curves. n = 3. (a) Resin 6 and variants. (b) Resin 7 and variants.


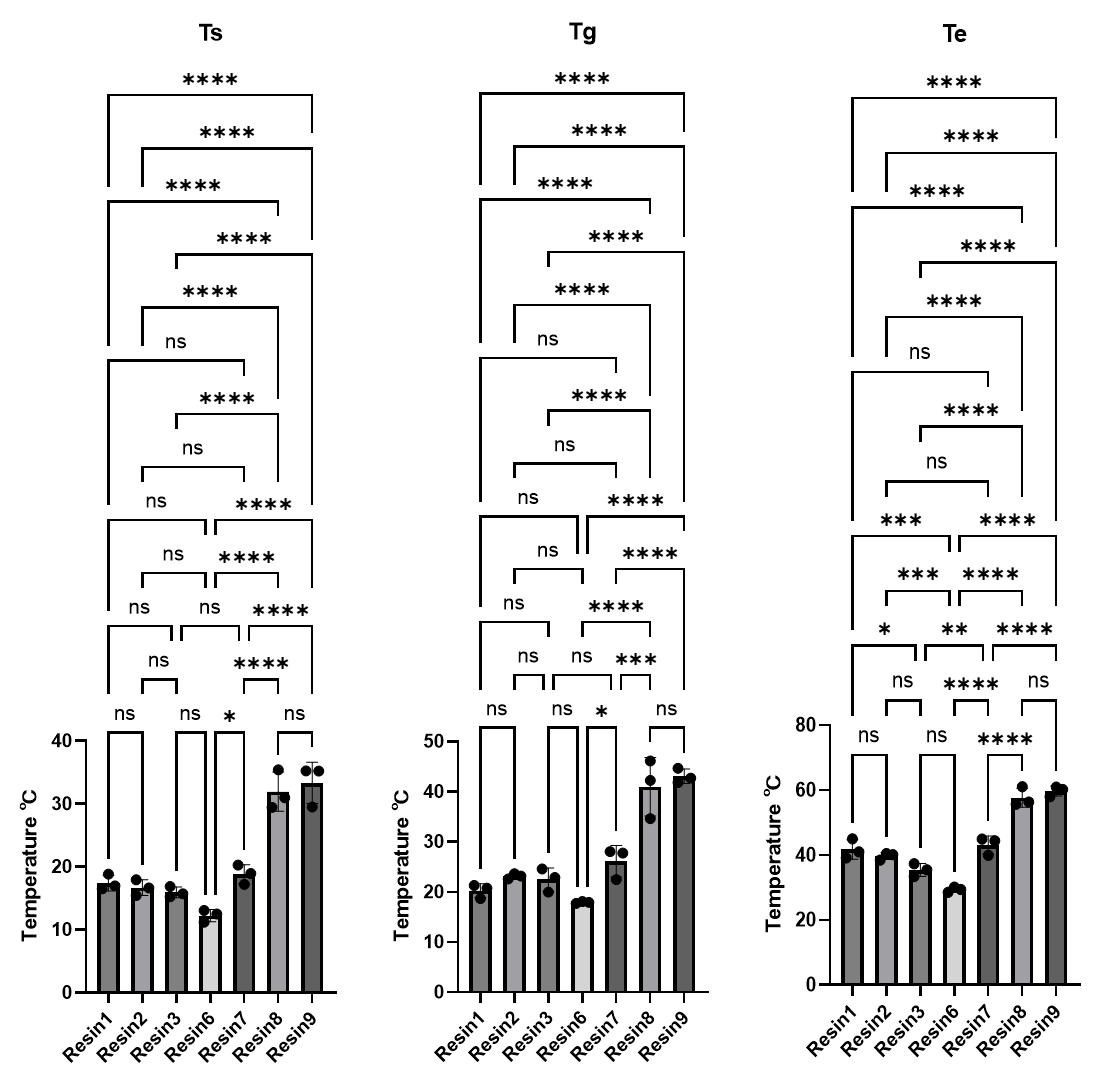


**Figure S4. Statistical comparison of key glass transition parameters among different resin formulations.** Statistical analysis of the onset temperature of glass transition (*T*_s_), glass transition temperature (*T*_g_), and end temperature of glass transition (*T*_e_), respectively. Statistical significance among groups was calculated using one-way ANOVA in GraphPad Prism. Error bars represent standard deviation (n = 3). These results indicate that formulations based on resins 8 and 9 exhibit significantly higher transition temperatures compared with other formulations, supporting their selection for subsequent material fabrication and performance evaluation.

Note: The glass transition behavior of the DSENO composites was evaluated via Differential Scanning Calorimetry (DSC). As shown in **Figure S1**, the transition from the glassy to the rubbery state manifests as a gradual step in heat capacity rather than a sharp inflection. This broadening of the *T*_g_ region is attributed to the restricted segmental mobility and increased heterogeneity inherent in the densely crosslinked TATATO/TMTMP network and the incorporation of Fe_3_O_4_ nanoparticles.^1,2^ To validate the differences in thermal properties, characteristic temperatures (*T*_s_, *T*_g_, and *T*_e_) were statistically analyzed using a one-way ANOVA (**Figure S4**). The results indicate that Resins 8 and 9 possess significantly higher transition temperatures (p < 0.05) compared to lower-numbered formulations. These findings confirm that the chemical composition significantly influences the network’s thermal stability, justifying the selection of these formulations for further development as vascular stents.

**Table S2.** Material candidate list


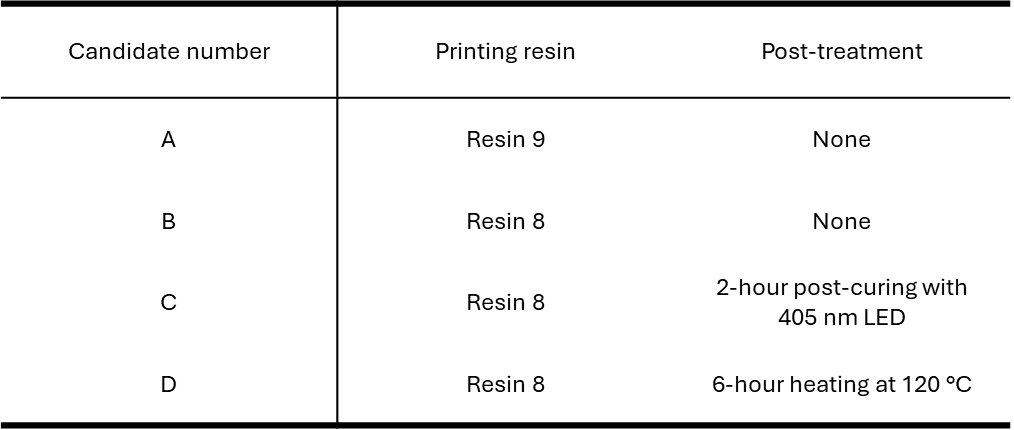


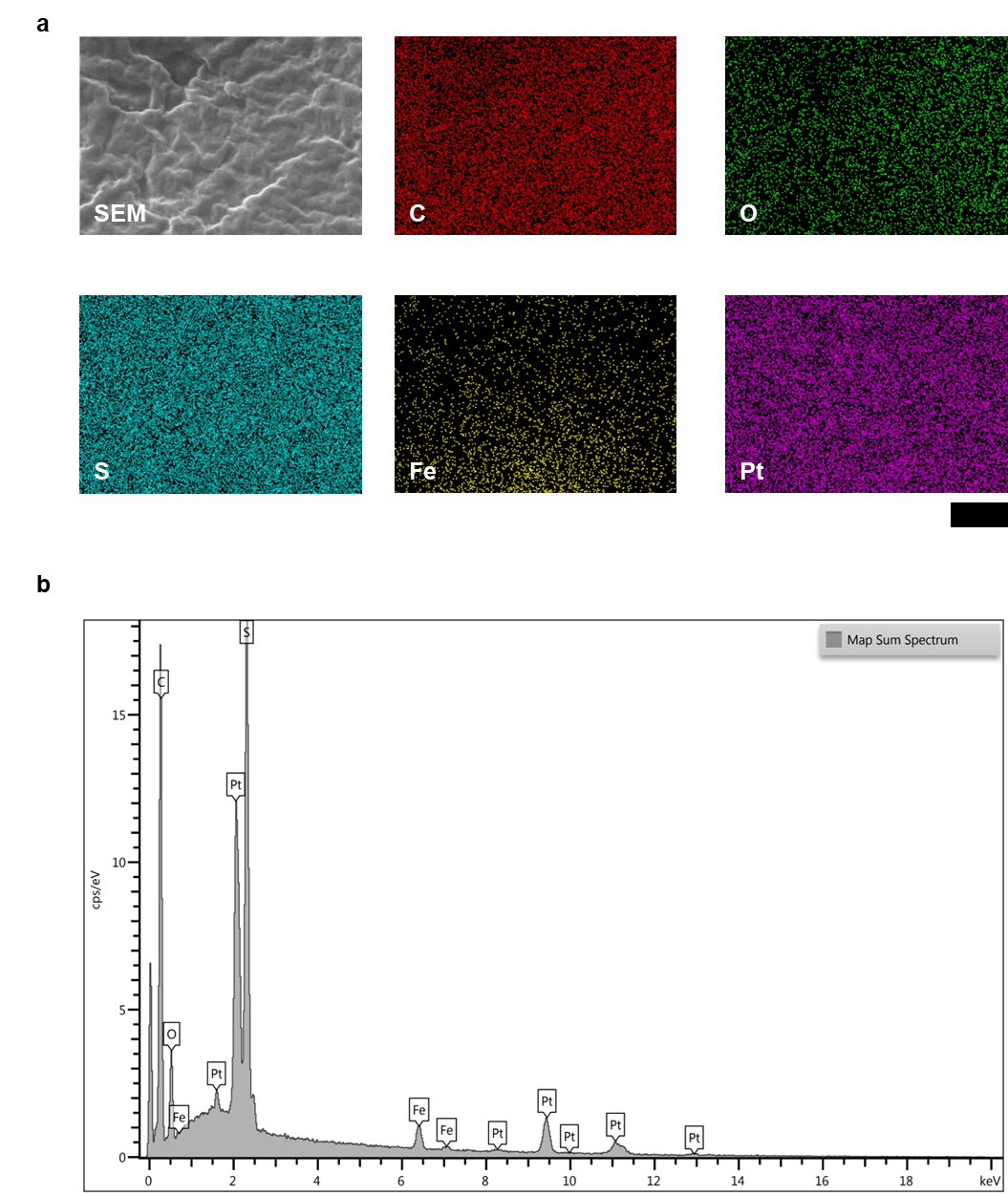


**Figure S5.** EDS data of element mapping images and spectrum for printed material homogeneity check, and the sample was coated with platinum. (a) Element mapping images with multiple elements mapping validating the homogeneity of the printed material. Scale bar = 1 µm. (b) Element spectrum.


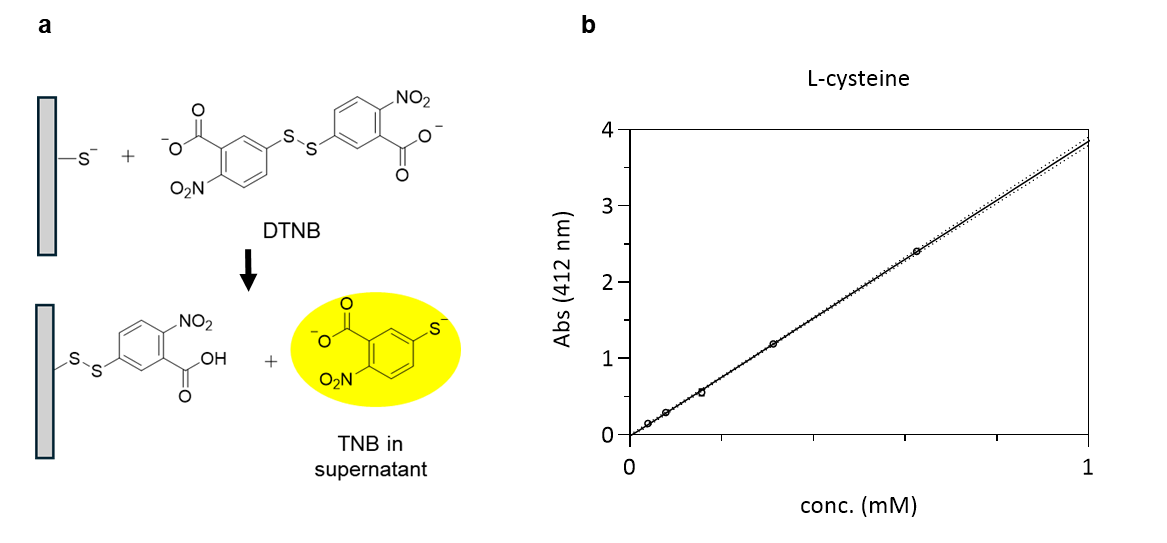


**Figure S6. Surface thiol detection.** (**a**) Schematics of surface thiol quantification based on Ellman’s assay. (**b**) Standard curve generated from L-cysteine.


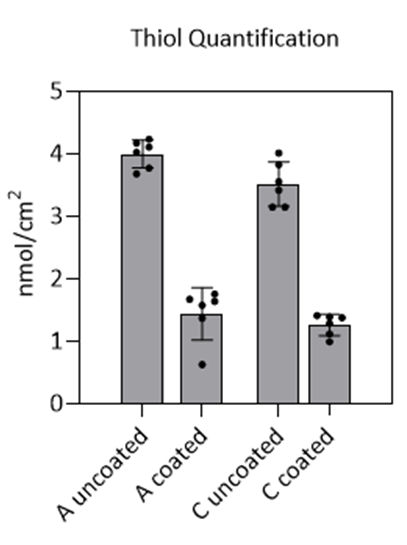


**Figure S7.** Thiol quantification before and after PEI coating of material A and C. n = 6.


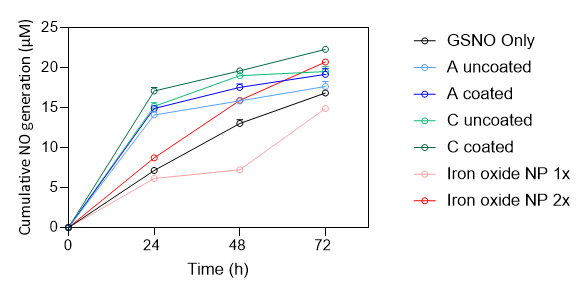


**Figure S8. Kinetics of Catalytic Nitric Oxide Generation.** Time-dependent cumulative NO release profiles under various experimental conditions. The curves demonstrate the sustained catalytic decomposition of S-nitrosothiols (RSNOs) over an extended duration, highlighting the steady-state flux achieved by the PEI-functionalized DSENO surfaces.

**Table S3.** Analysed XPS peaks and the corresponding chemical bonds.


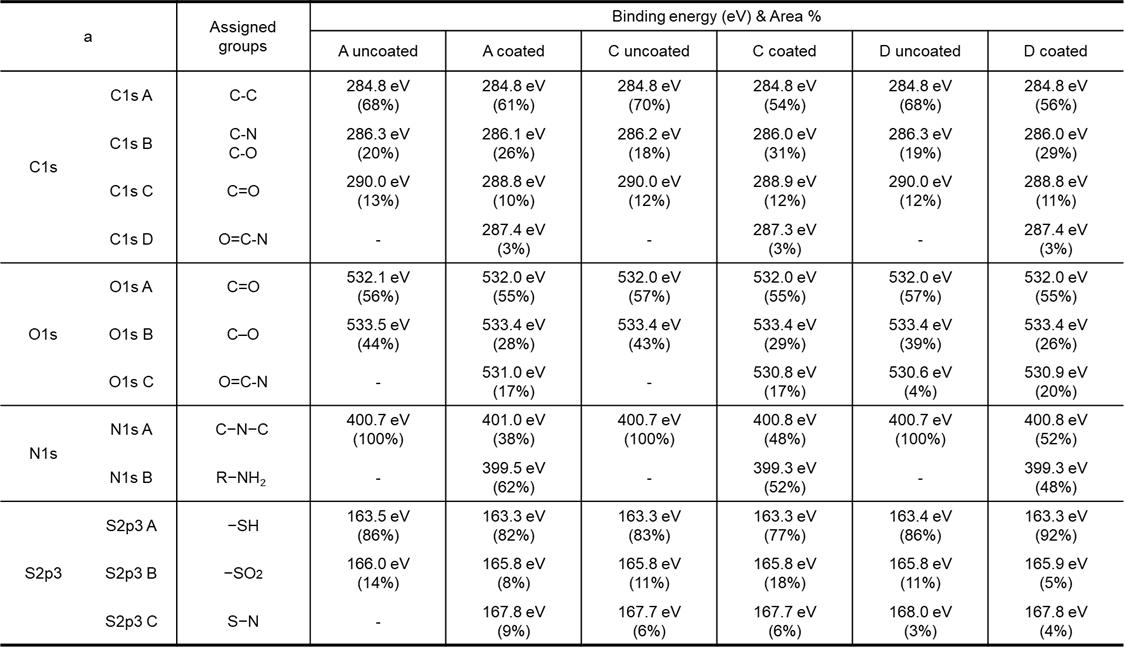


**Table S4.** Atomic percentage of C, N, O, and S on uncoated and coated materials.


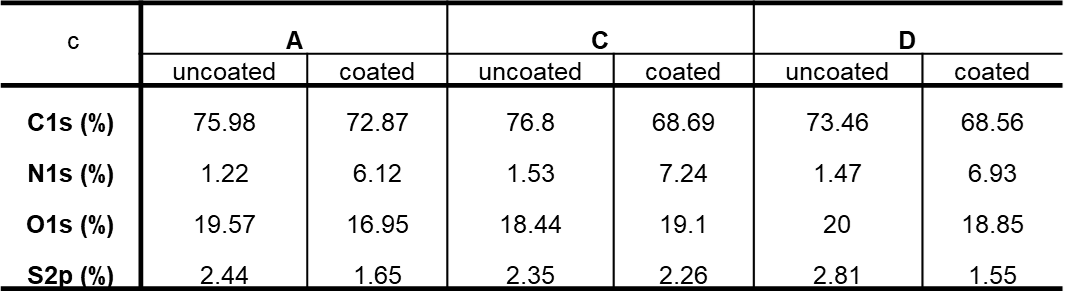


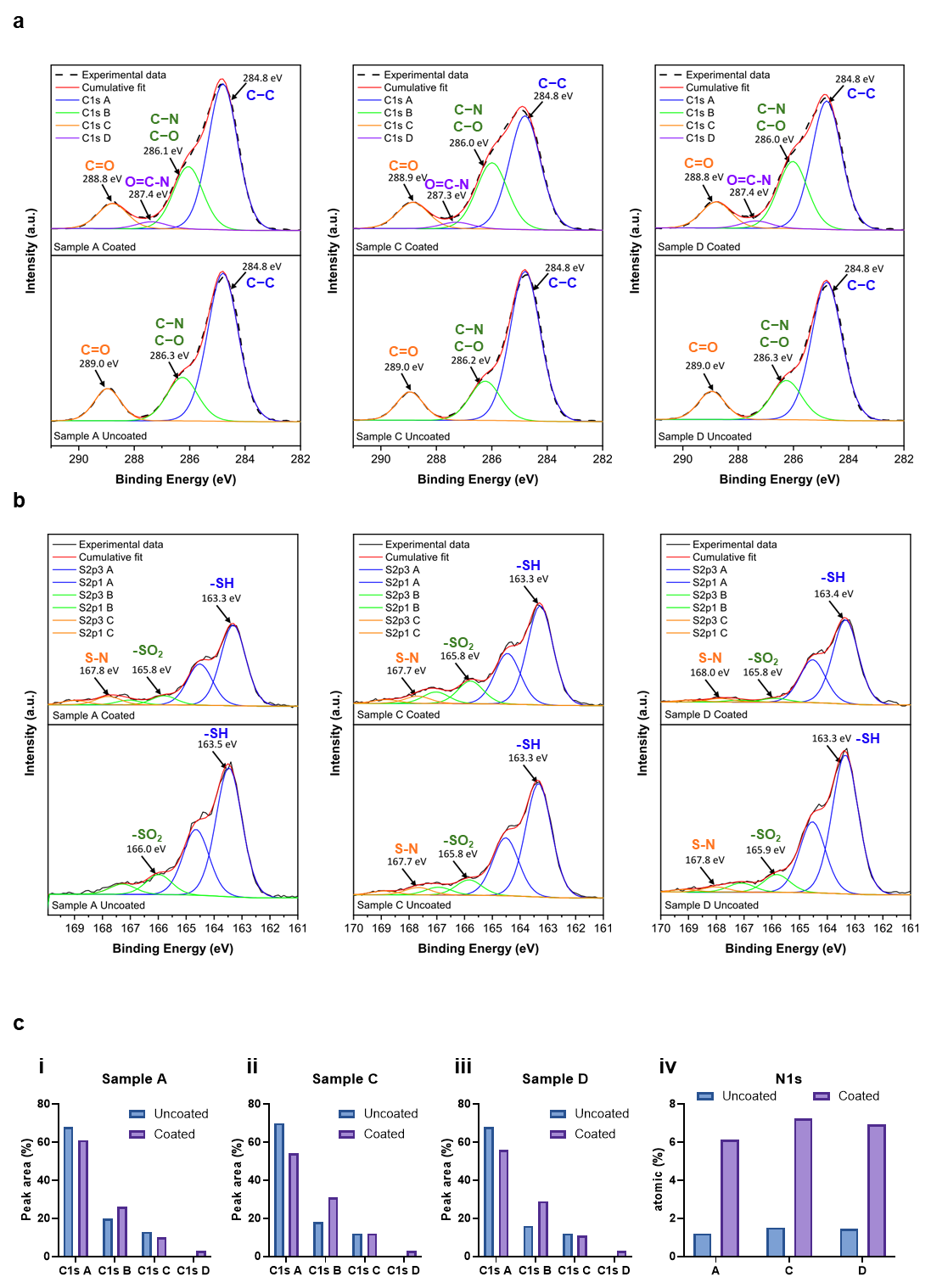


**Figure S9. Compositional analysis of coated and uncoated candidate material A, C and D groups with XPS.** (**a**) High resolution C1s spectra. (**b**) High resolution S2p spectra. (**c**) (**i**) (**ii**) (**ii**) Peak area ratios of coated and uncoated material A, C, D in C1s assignment, and (**iv**) The surface nitrogen atomic ratio of coated and uncoated material A, C, D.


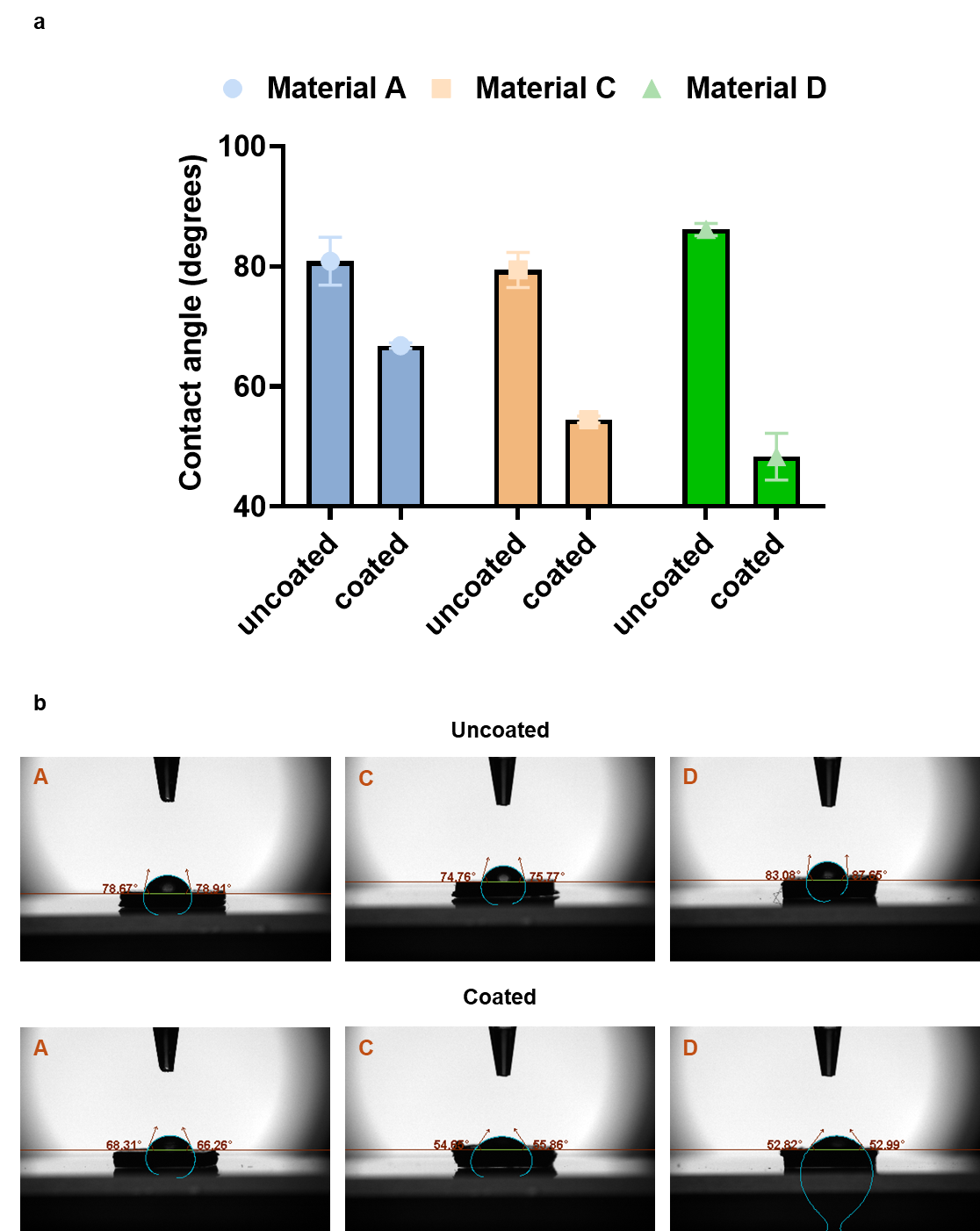


**Figure S10. Water contact angle tests of uncoated and coated material A, C, and D, and the coating of PEI improved the hydrophilicity of material surfaces. n = 3**. (**a**) Contact angle data comparison. (**b**) Screenshots of water contact angle measurements.


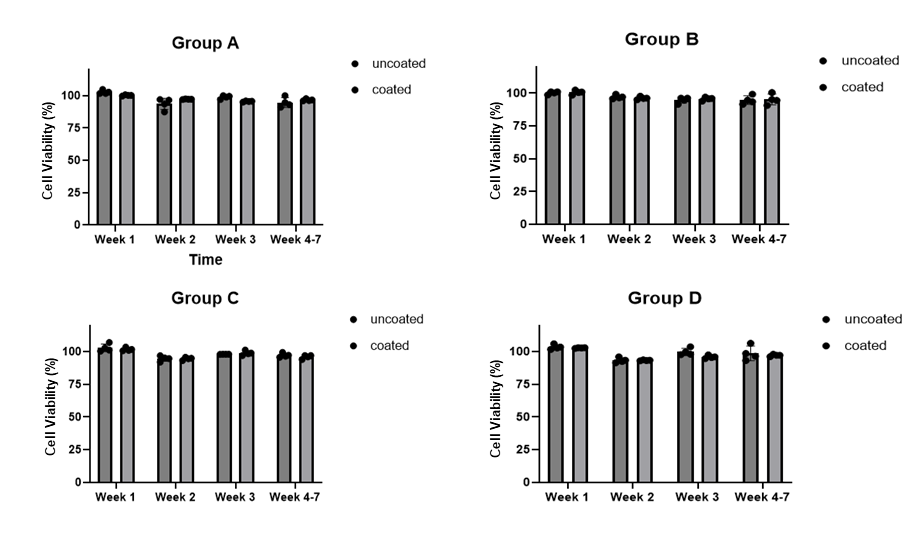


**Figure S11.** **Extract-based cytotoxicity evaluation of printed materials.** Uncoated and PEI-coated materials A, B, C, and D were incubated in cell culture medium (EGM-2 MV) for multiple weeks to obtain conditioned media. The conditioned media were then applied to HUVECs, and cell viability was evaluated after 48 hours of culture. n = 3.


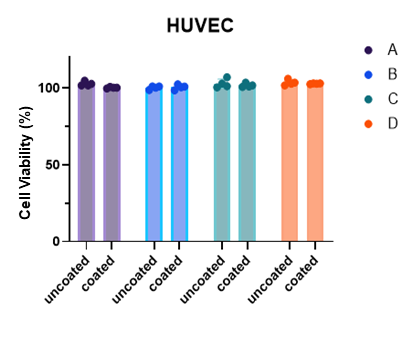


**Figure S12.** Biocompatibility evaluation using direct HUVEC–material contact. n = 4.


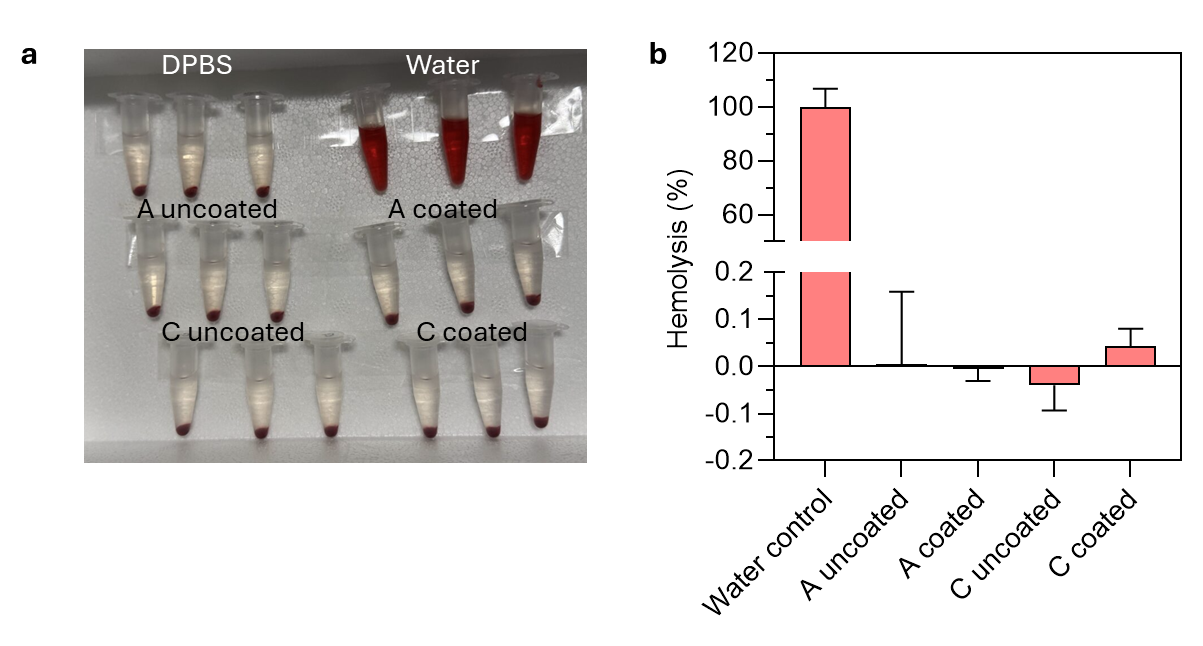


**Figure S13. Hemolysis and Hemocompatibility Assessment.** (a) Representative photographic results of red blood cell (RBC) supernatants following incubation with the DSENO stent and controls. (b) Quantitative evaluation of the hemolysis percentage under different experimental conditions. Data are presented as mean ± SD (n = 3).

**
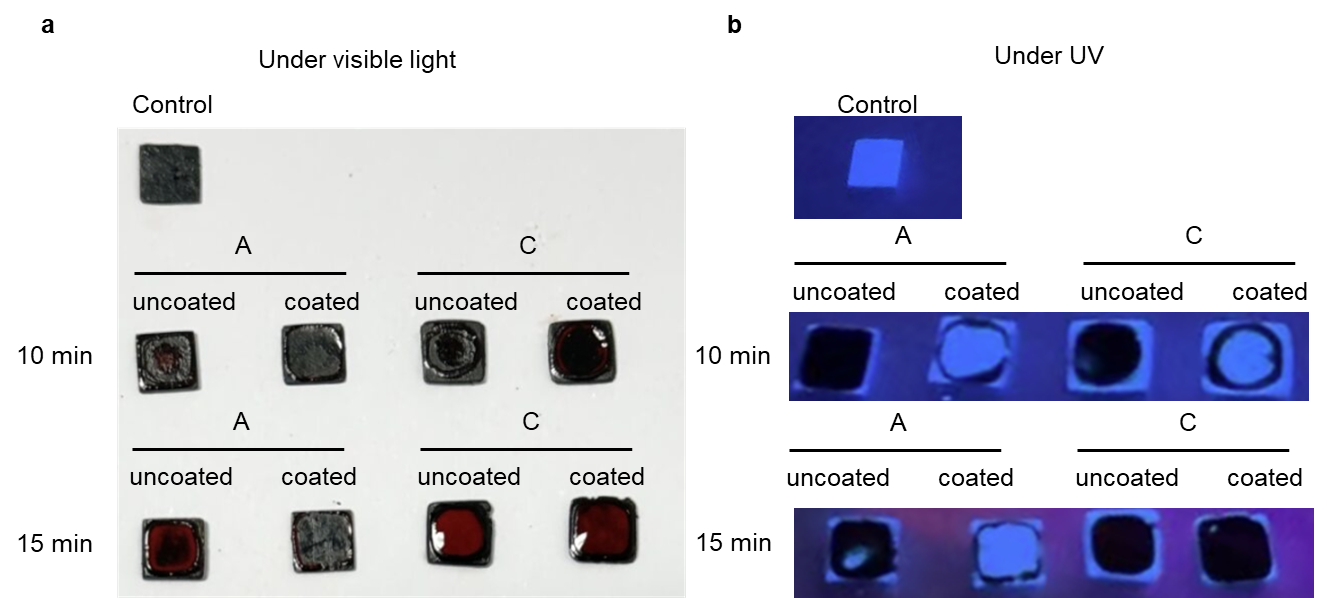
**

**Figure S14**. **Whole blood coagulation behavior on coated and uncoated 3D-printed samples. Representative images of samples after incubation with recalcified whole blood at 37 °C for 10 and 15 min.** (**a**) Photographs acquired under visible light showing macroscopic clot formation on uncoated and coated samples from A and C. (**b**) Corresponding images acquired under UV illumination to enhance contrast and visualize retained blood clots on the dark material surfaces.


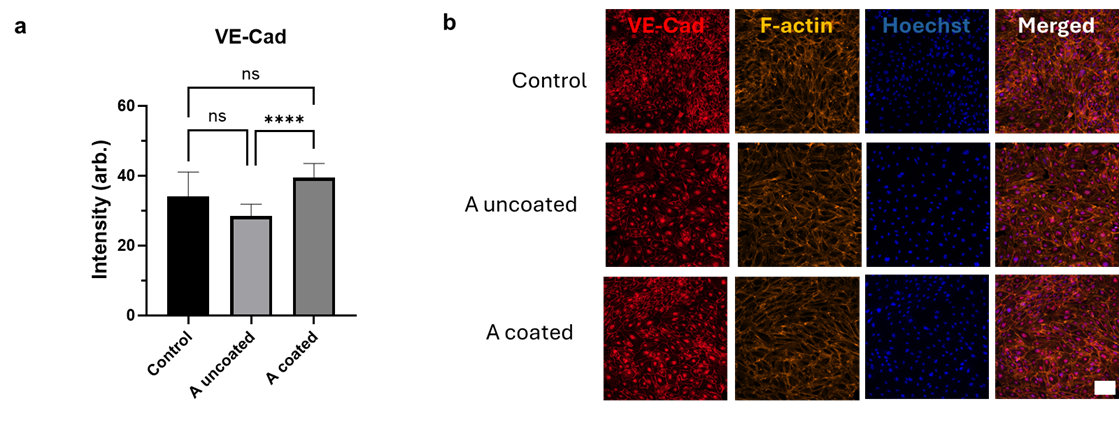


**Figure S15. Evaluation of endothelial cell junction integrity and functional phenotype by VE-cadherin staining**. (**a**) Quantitative analysis of VE-cadherin fluorescence intensity. Statistical significance was calculated using one way ANOVA, error bars represent standard deviation. n = 3. ROI > = 6. (**b**) Representative immunofluorescence images of HUVECs control, with uncoated material A, and with PEI-coated material A. VE-cadherin (red) indicates endothelial cell–cell junctions, F-actin (orange) shows cytoskeletal organization, and nuclei were counterstained with Hoechst (blue). Merged images demonstrate overall cell morphology and junctional continuity. Scale bar = 100 µm.


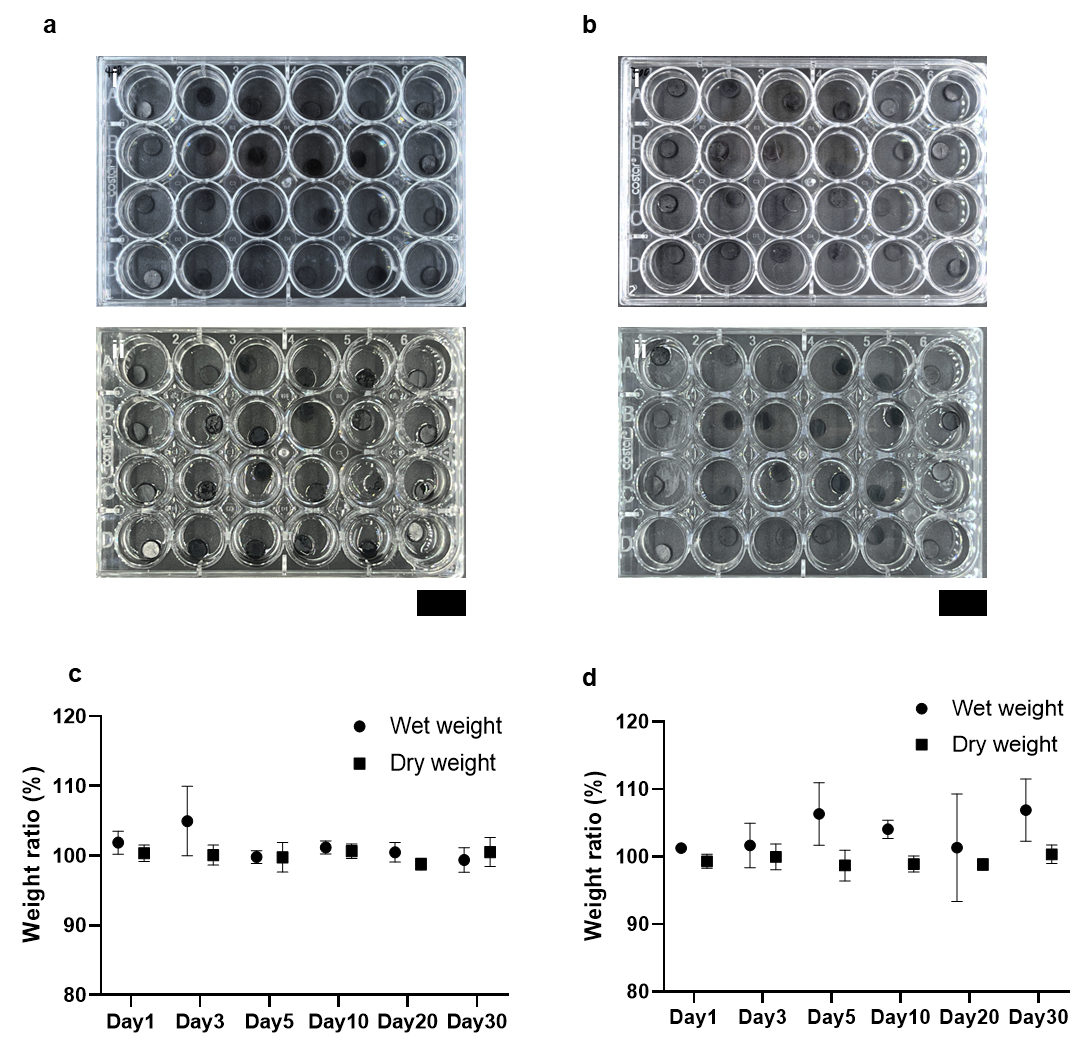


**Figure S16. Material incubation for weight change test. Scale bar = 15 mm.** (**a**) Photos of material candidate A before (i) and after (ii) incubation without visible changes. (**b**) Photos of material candidate B before (i) and after (ii) incubation without visible changes. (**c**) Wet weight and dry weight comparison at different time points of material candidate A, demonstrating the negligible swelling. (**d**) Wet weight and dry weight comparison at different time points of material candidate B, and compared to material A, material B showed slightly swelled due to the incubation.


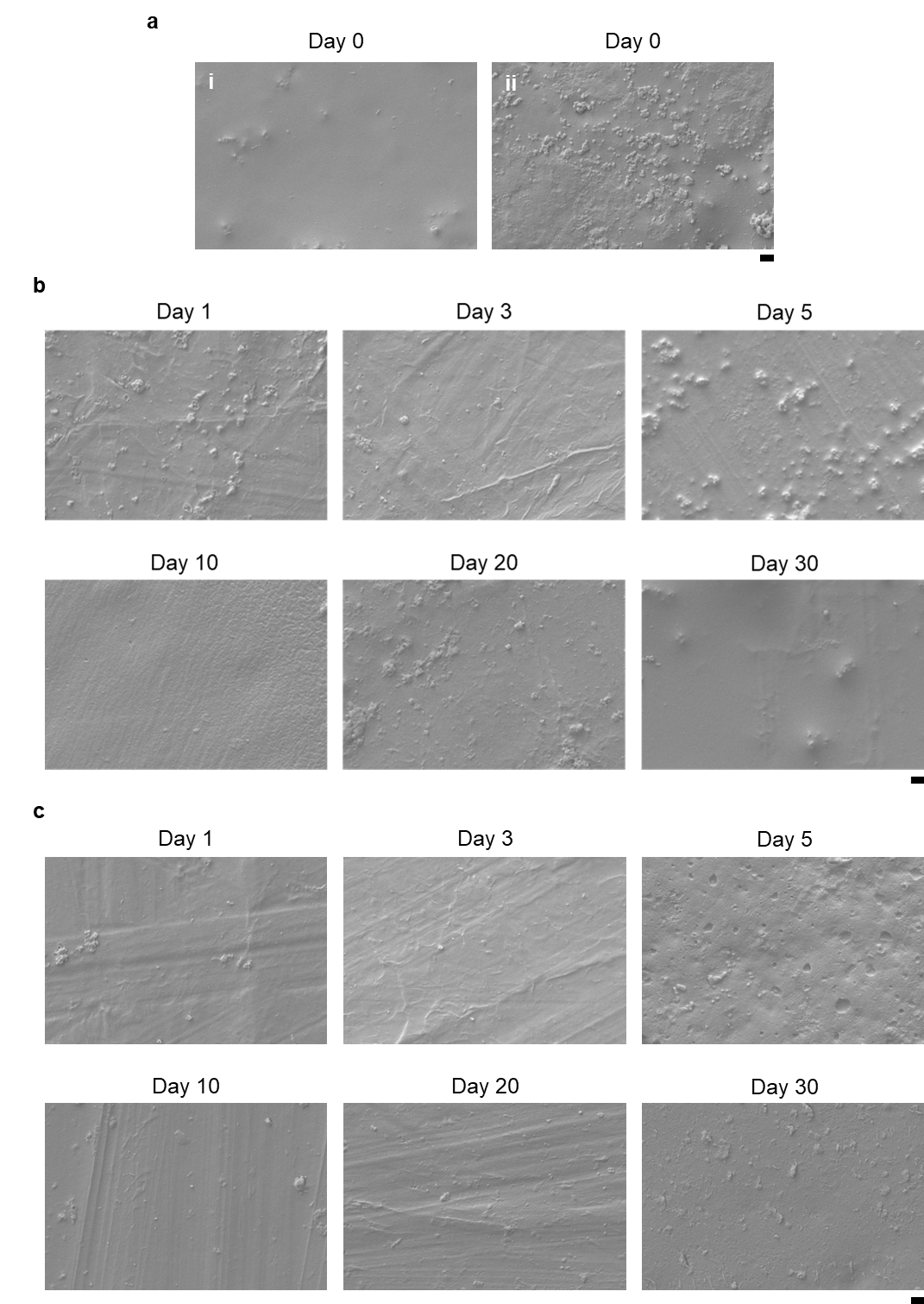


**Figure S17. SEM images of material incubation samples to check the surface integrity and porosity. Scale bar = 1 µm.** (**a**) Images of material candidate A (i) and B (ii) at day 0 with solid and intact surfaces. (**b**) Images of material candidate A at different time points, where the material surfaces showed no changes within one month incubation, validating the material stability. (**c**) Images of material candidate B at the same time points, and the surfaces had no pores.


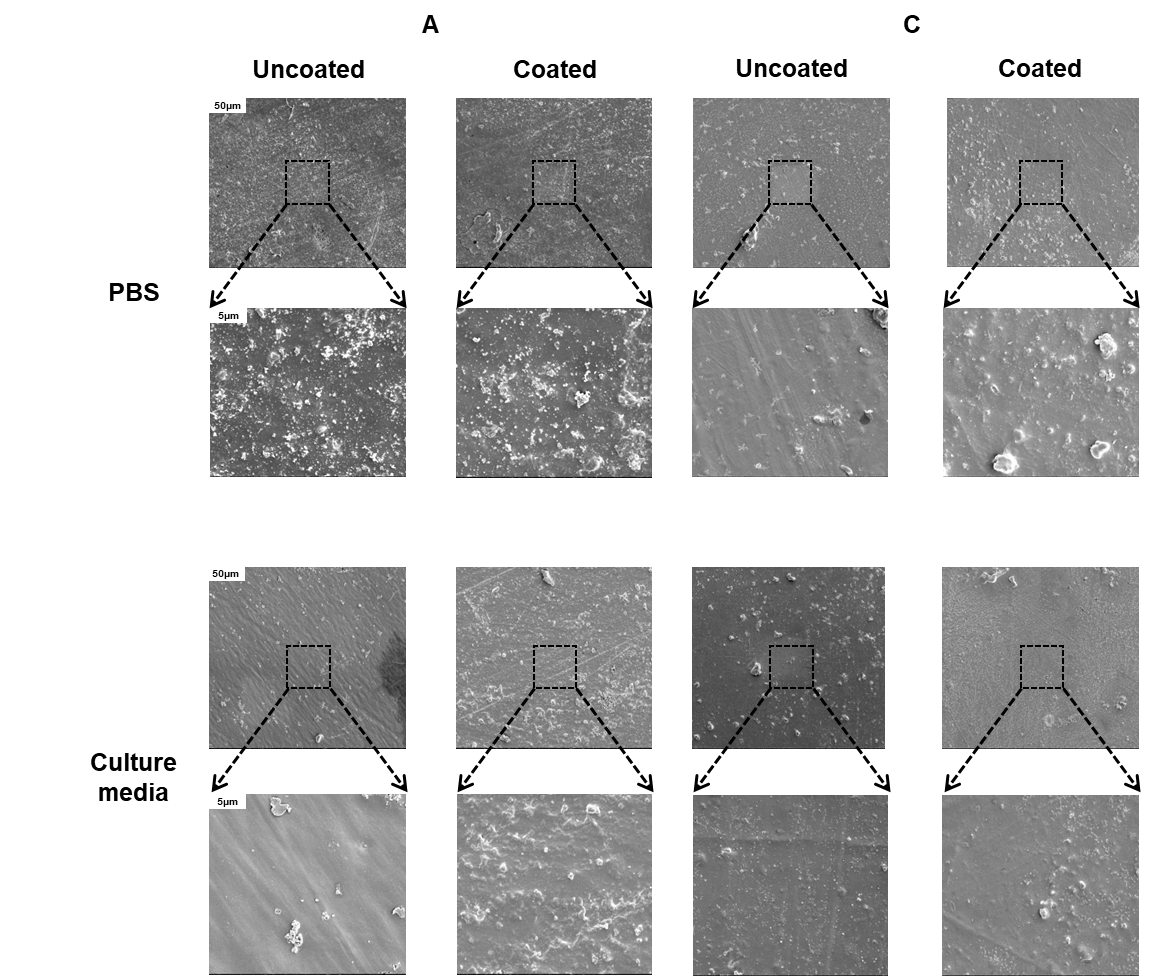


**Figure S18. Long-term Morphological Stability and Surface Integrity**. Representative scanning electron microscopy (SEM) micrographs of uncoated and PEI-coated scaffolds (formulations A and C) following a 60-day incubation period in phosphate-buffered saline (PBS) and cell culture medium. The images demonstrate the structural persistence of the 3D-printed architecture and the stability of the catalytic coating under simulated physiological conditions. Scale bars represent 50 µm (low magnification) and 5 µm (high magnification).


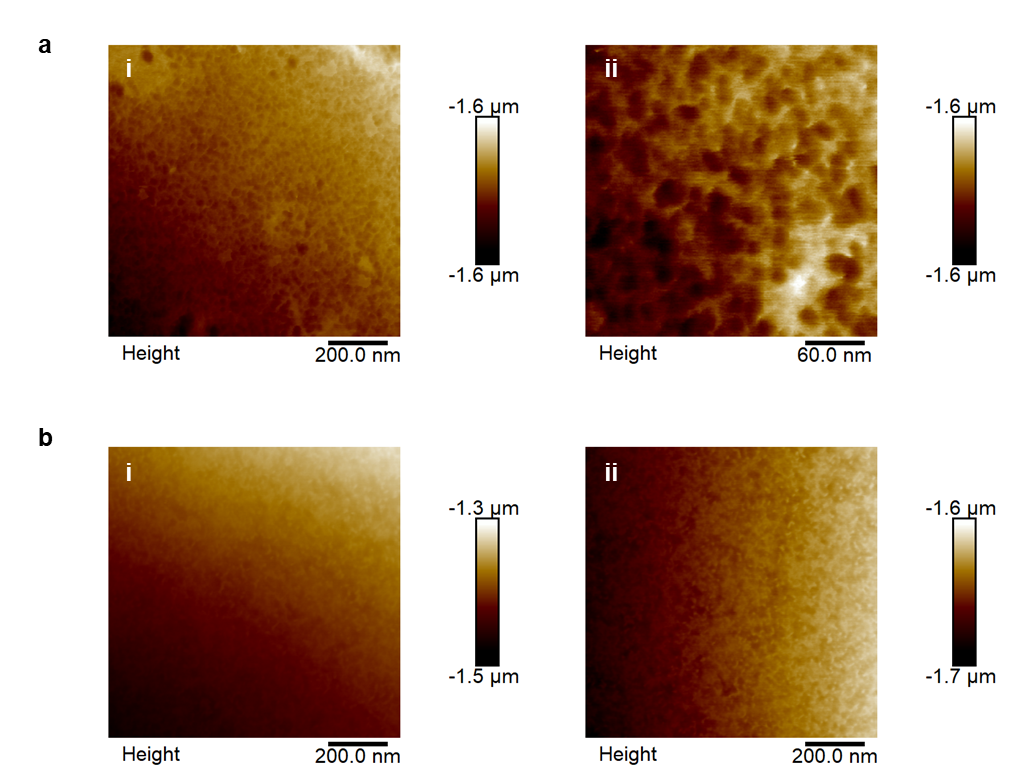


**Figure S19. AFM images of surface morphology.** Representative 2D AFM images and corresponding height maps for (**a**) Sample 1 and (**b**) Sample 2. Surface roughness parameters were evaluated at two distinct scanned spots (labelled **i** and **ii**) for each sample. Roughness values for Sample 1: (**i**) Ra = 1.40 nm, Rq = 1.89 nm; (**ii**) Ra = 0.94 nm, Rq = 1.21 nm. Roughness values for Sample 2: (**i**) Ra = 2.60 nm, Rq = 3.20 nm; (**ii**) Ra = 1.53 nm, Rq = 1.89 nm. Ra (arithmetic average roughness) and Rq (root mean square roughness) were calculated using NanoScope Analysis software (Bruker) according to standard definitions. Scale bar = 200 nm.


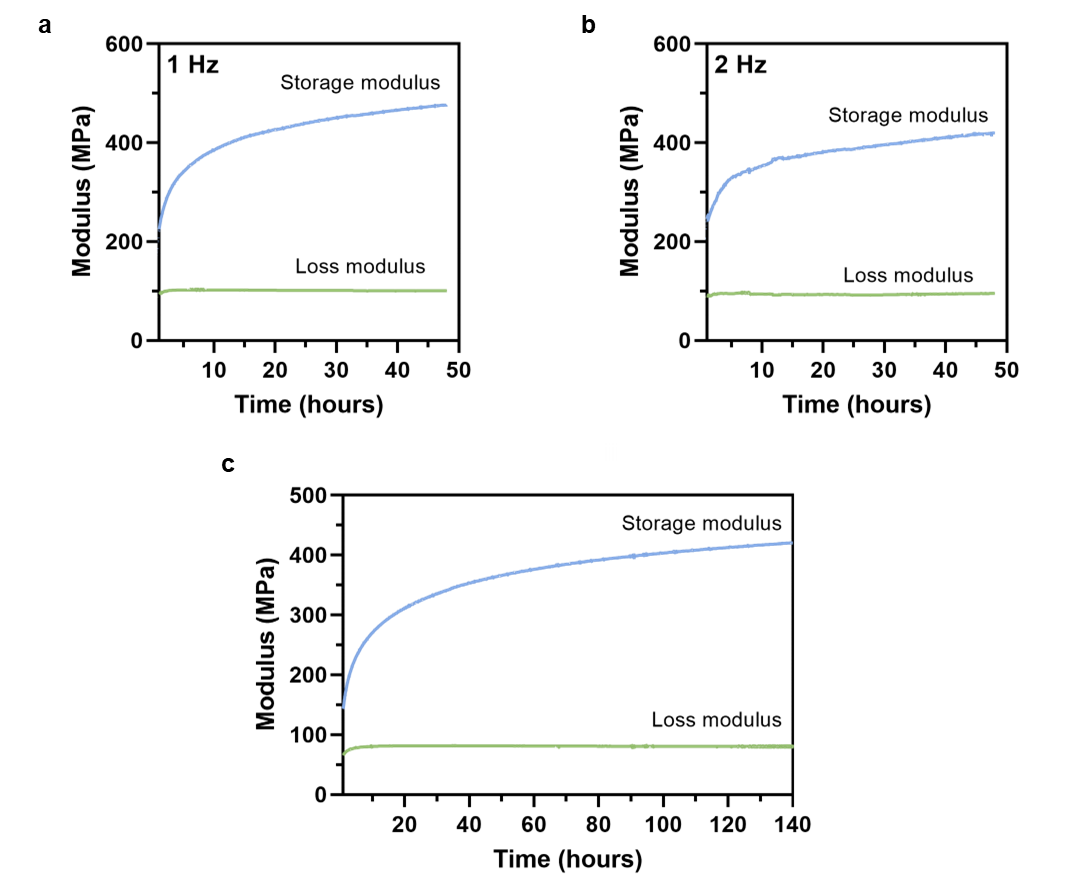


**Figure S20. Extended DMA test results.** (**a**) 1 Hz 48 hours test. (**b**) 2 Hz 48 hours test. (**c**) 2 Hz 140 hours test.


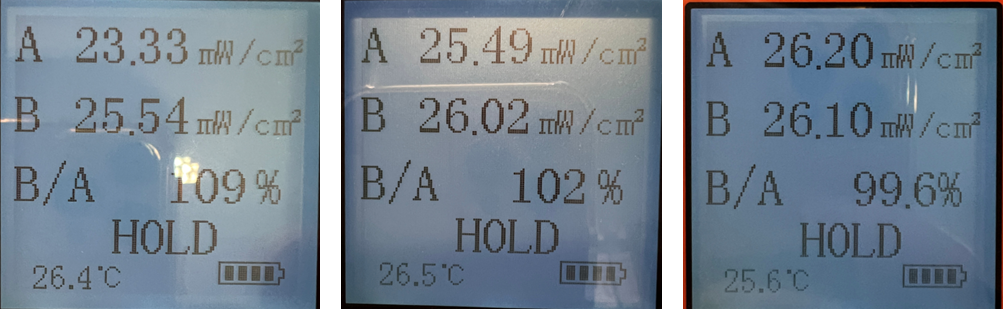


**Figure S21.** Light intensity measurement with three repeats.


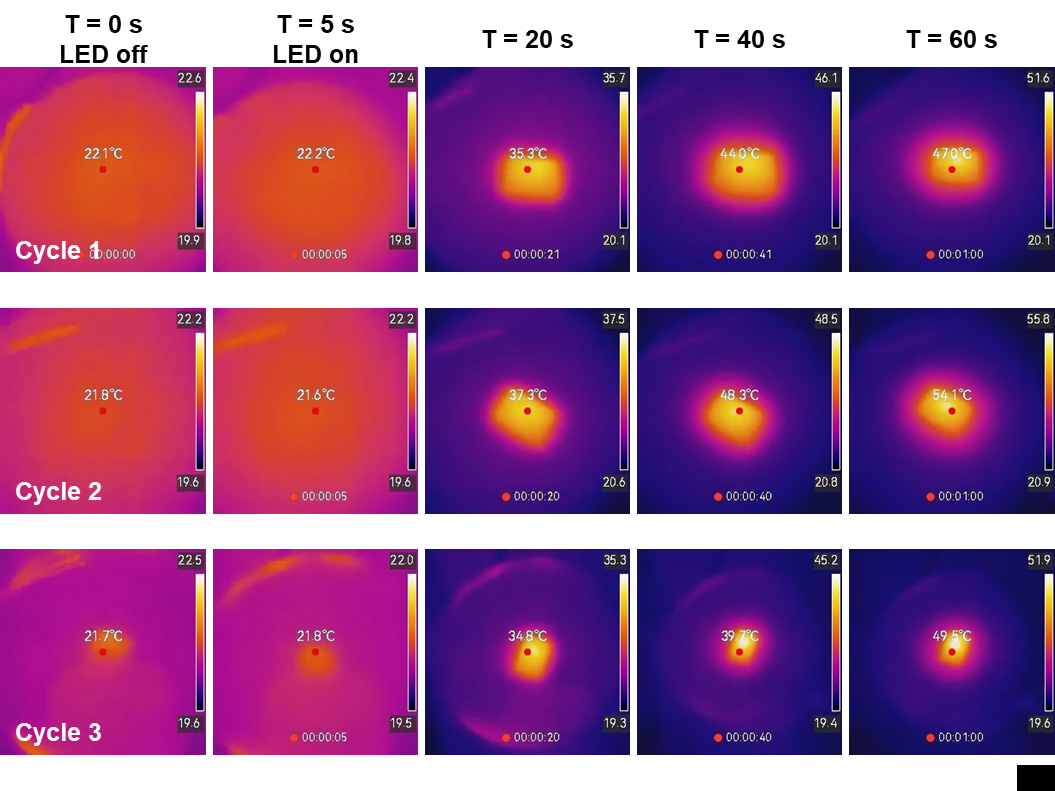


**Figure S22.** Cyclic NIR heating tests for the same sample. Scale bar = 5 mm.

**Figure S23.** Cyclic NIR heating temperature ramp curves for the same sample.


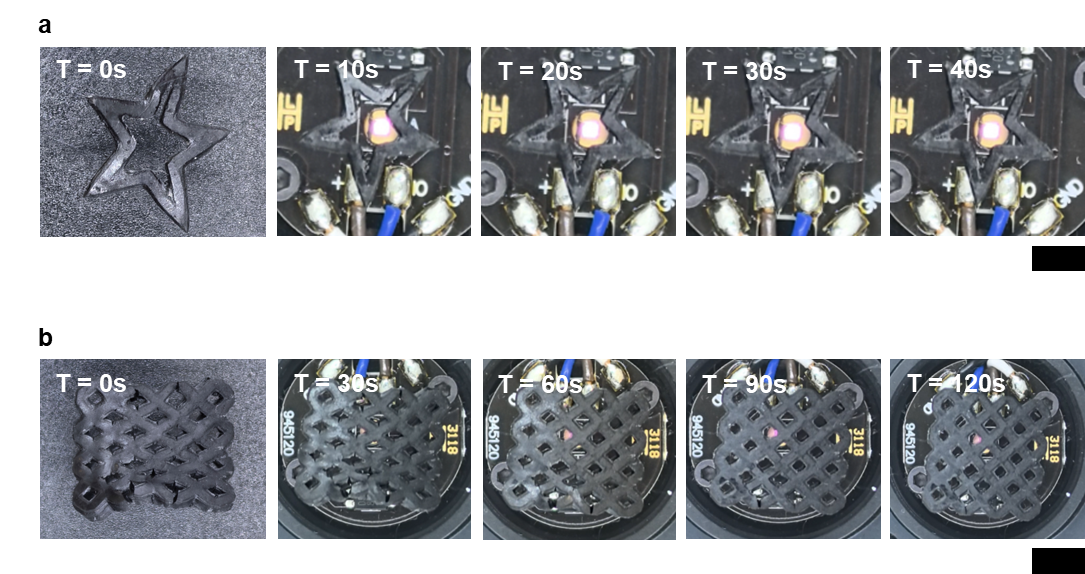


**Figure S24. Photos of NIR activated shape recovery. Scale bar = 2.5 mm.** (**a**) Star shape (**b**) 16-repeat unit porous structure.


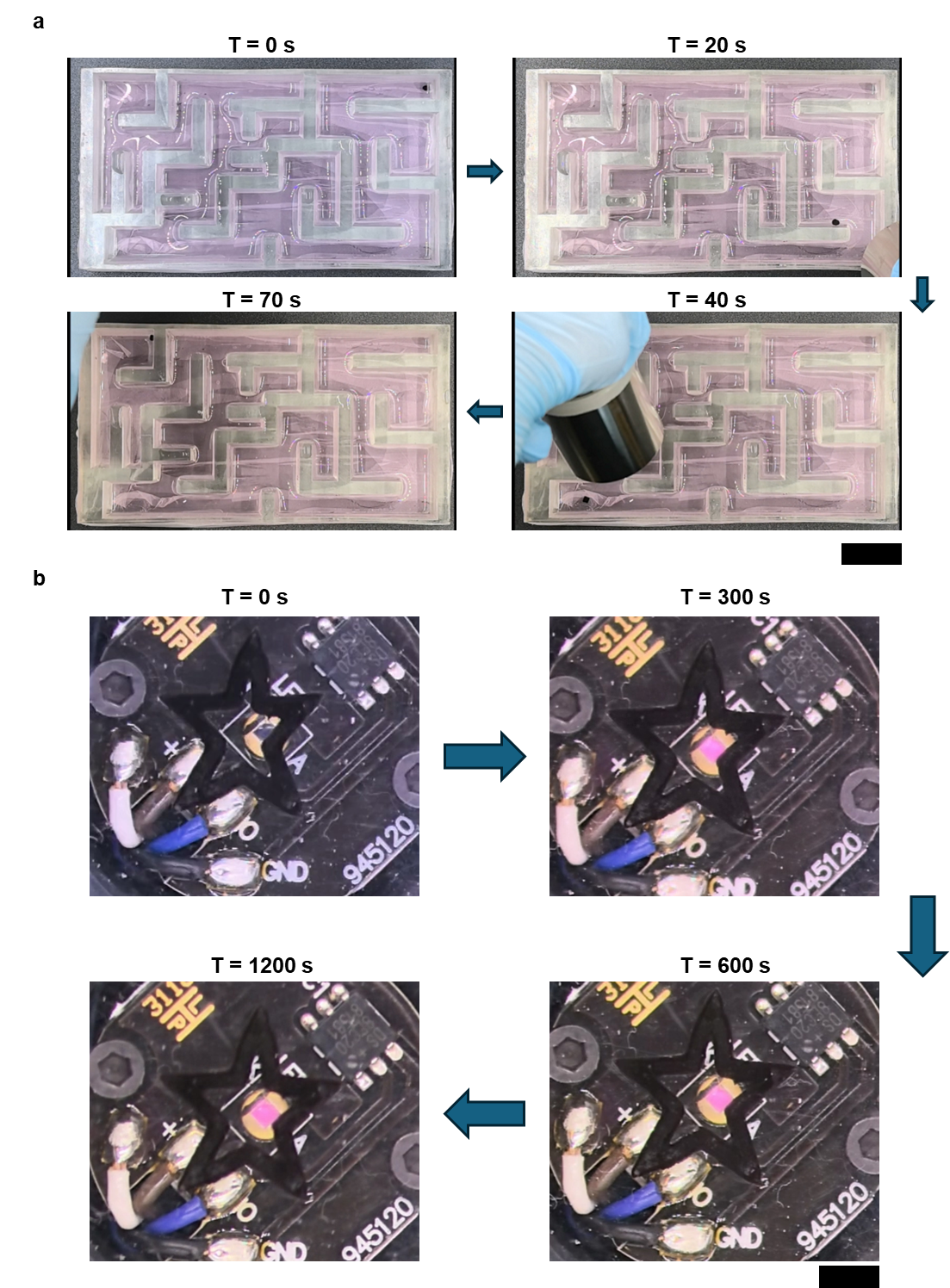


**Figure S25. Untethered control investigations in microvascular endothelial cell growth medium.** (**a**) Magnetic manipulation of a cylindrical structure navigating through a maze. Scale bar = 1.5 cm. (**b**) Photothermal activation demonstrated on the compressed star structure. Scale bar = 2 mm.

Note: As shown in **Figure S25a** and **Video S8**, the cylindrical structure exhibited excellent mobility, successfully navigating a complex maze via external magnetic manipulation. The increased viscosity and ionic strength of the medium did not hinder the magnetic responsiveness of the material. Additionally, the **photothermal shape-memory effect** was evaluated using NIR-LED activation. The compressed star structure achieved rapid and complete structural deployment within the medium (**Figure S25b, Video S9**).


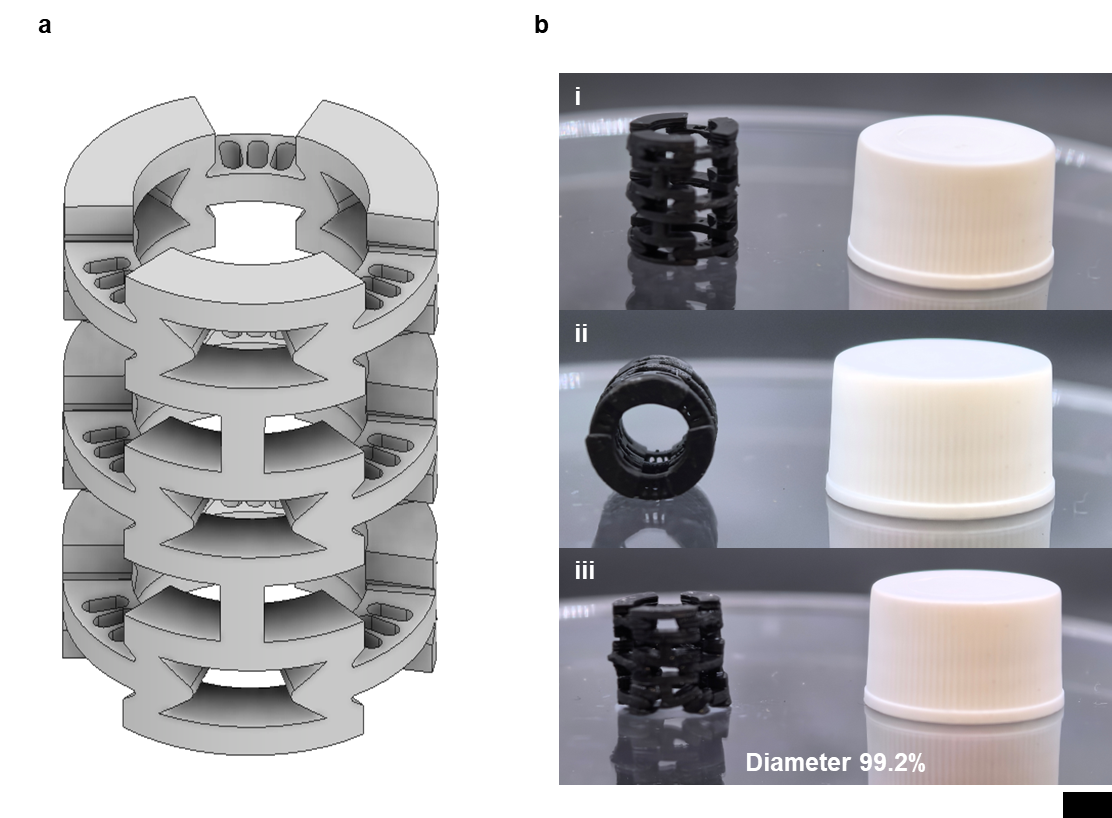


**Figure S26. Type I variant compression test. Scale bar = 5 mm.** (**a**) 3D model (**b**) Size change evaluation (i) (ii) Before compression. (iii) After compression.


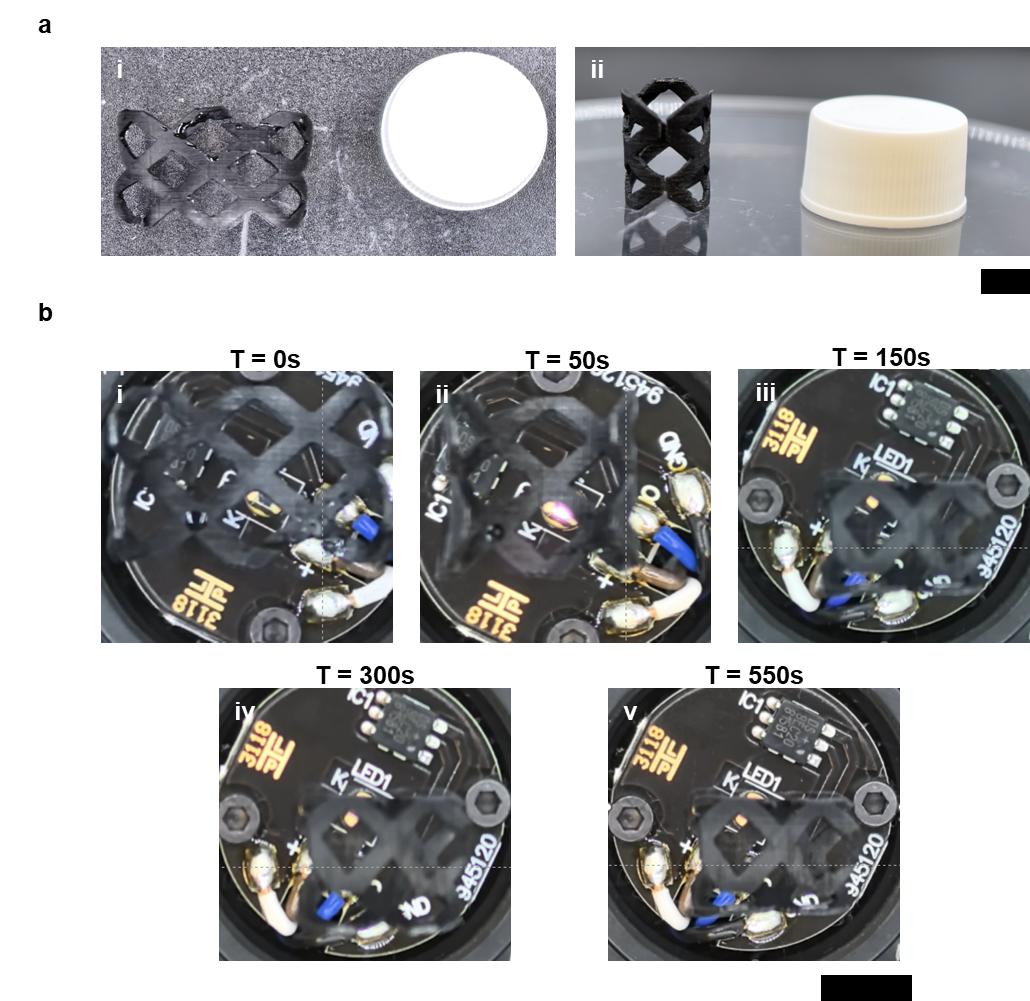


**Figure S27. Type II variant shape recovery after cutting and shape-morphing. Scale bar = 5 mm.** (**a**) (i) Temporary shape. (ii) Recovered shape. (**b**) Time lapse of NIR induced shape recovery.


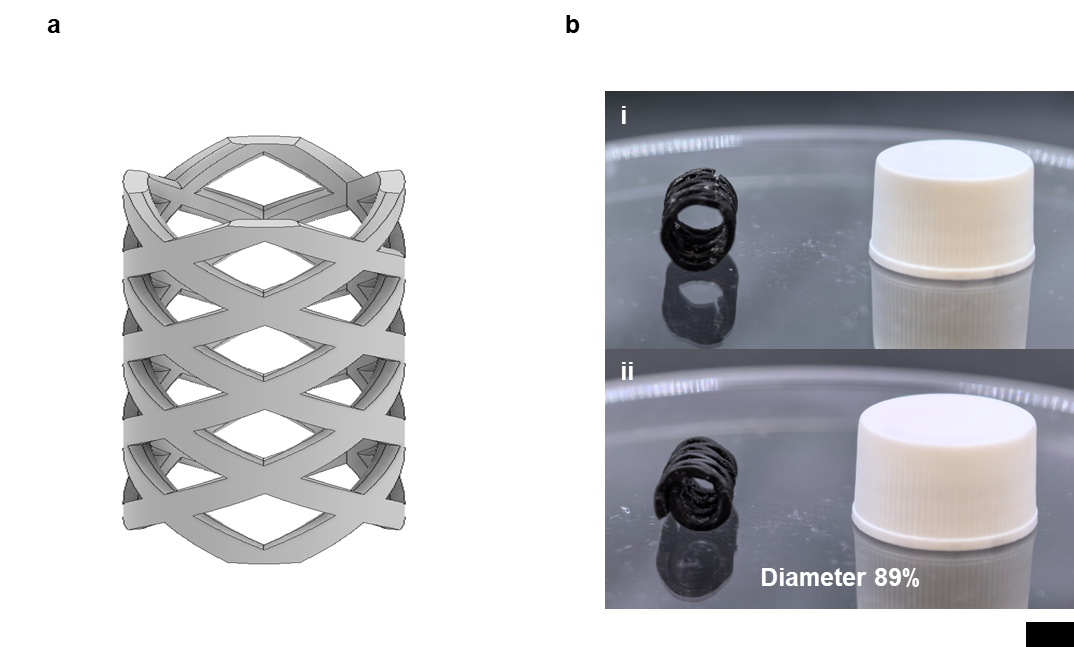


**Figure S28. Type III variant rolling test. Scale bar = 5 mm.** (**a**) 3D model (**b**) Size change evaluation (i) Before rolling. (ii) After rolling.


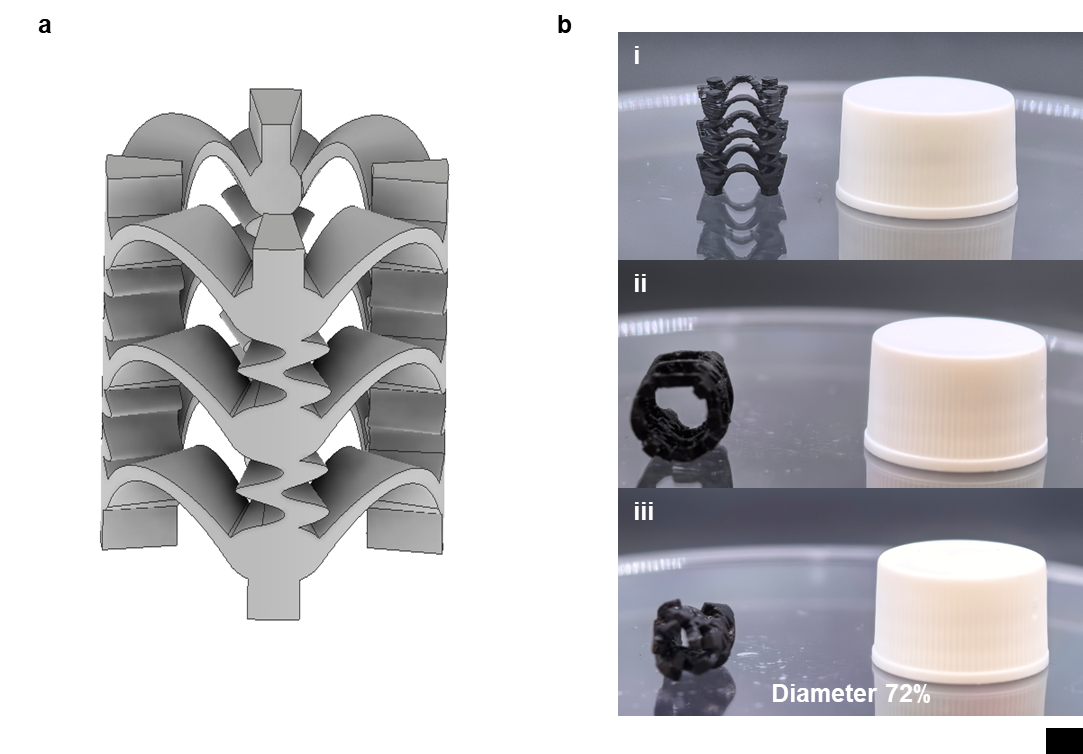


**Figure S29. Type IV compression test. Scale bar = 5 mm.** (**a**) 3D model (**b**) Size change evaluation (i) (ii) Before compression. (iii) After compression.


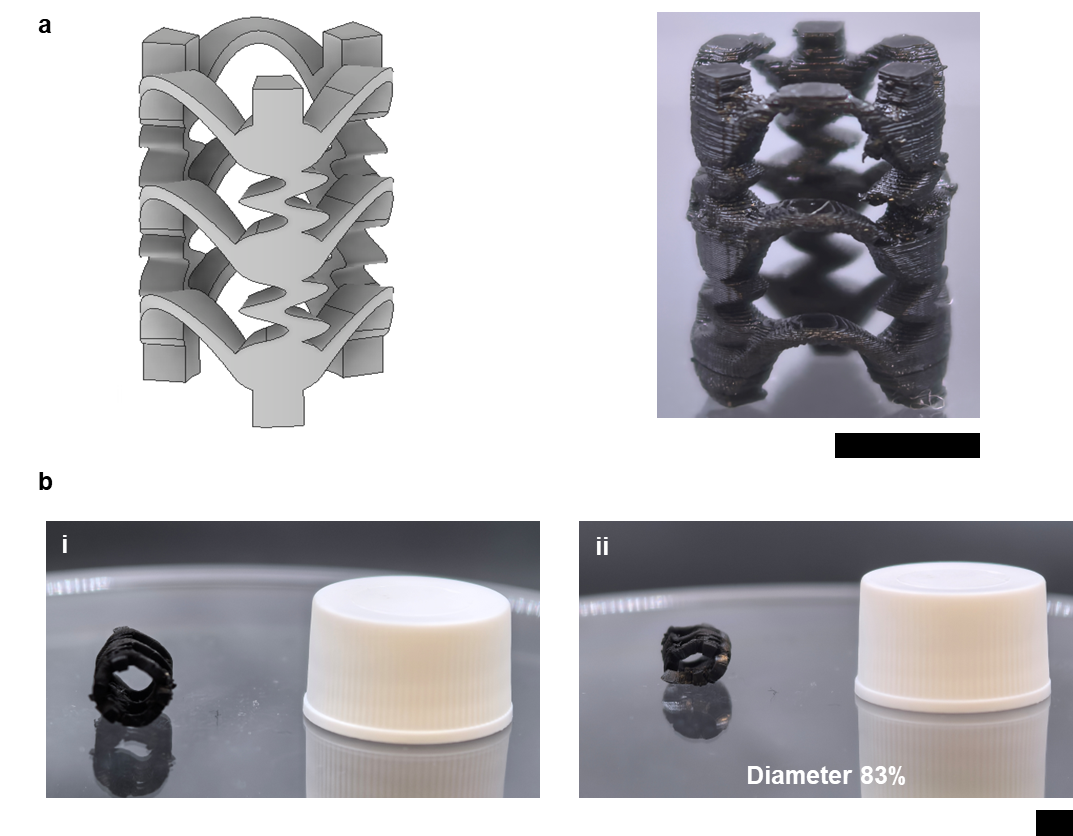


**Figure S30. Type IV variant compression test. Scale bar = 5 mm.** (**a**) 3D model and printed model. (**b**) Size change evaluation (i) Before compression. (ii) After compression.


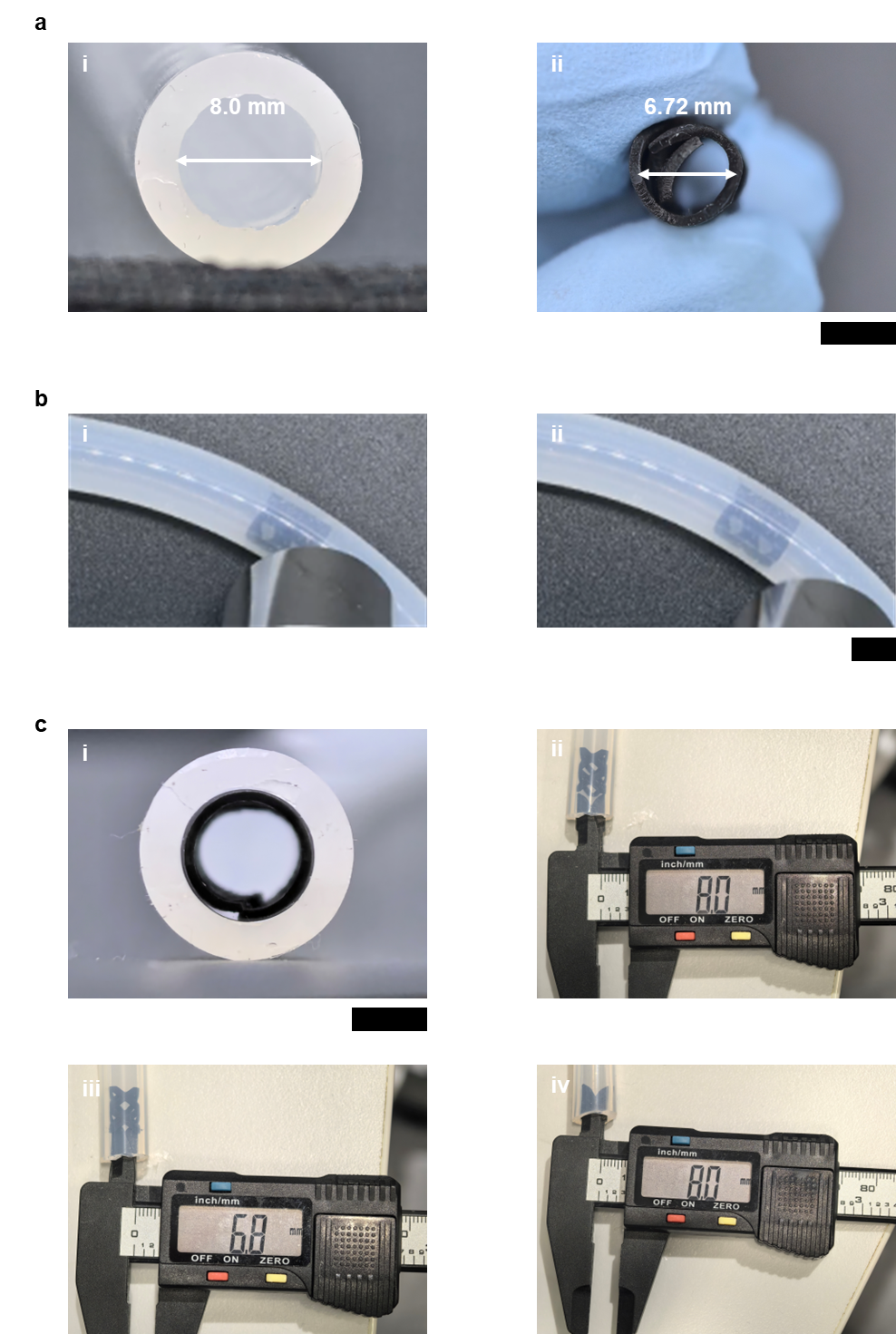


**Figure S31. Tube model photos and diameter measurement. Scale bar = 5 mm.** (**a**) (i) Cross-section of the tube. (ii) Compressed Type II DSENO image. (**b**) (i) (ii) Screenshots of magnetic controlled movement after deployment. (**b**) (i) Cross-section image after DSENO deployment. (ii) Measurement of the closer side inner diameter of the tube. (iii) Measurement of the hollow diameter of the stent. (iv) Measurement of the rare side inner diameter of the tube.

**Legends of supplementary Videos**

**Video S1**. Magnetically controlled movement in maze.

**Video S2.** NIR photo-thermal induced shape recovery of 6 repeat unit structure.

**Video S3.** NIR photo-thermal induced shape recovery of 16 repeat unit structure.

**Video S4.** NIR photo-thermal induced shape recovery of star shape structure.

**Video S5.** NIR photo-thermal induced shape recovery of Type II DSENO stent.

**Video S6.** Magnetically controlled movement in tube model before and after shape recovery.

**Video S7.** NIR photo-thermal induced deployment of Type II DSENO stent in tube model.

**Video S8.** Magnetically controlled movement in maze with cell culture media.

**Video S9.** NIR photo-thermal induced shape recovery of star shape structure with cell culture media.

**Video S10.** Magnetically controlled movement in vessel model.

**References:**

1 Lin, C.-L. & Chiu, W.-Y. Thermally responsive complex polymer networks containing Fe3O4 nanoparticles: Composition/morphology/property relationship. *Journal of Polymer Science Part A: Polymer Chemistry* **43**, 5923-5934 (2005). <https://doi.org/https://doi.org/10.1002/pola.21079>.

2 Yakacki, C. M., Satarkar, N. S., Gall, K., Likos, R. & Hilt, J. Z. Shape-memory polymer networks with Fe3O4 nanoparticles for remote activation. *J Appl Polym Sci* **112**, 3166-3176 (2009). <https://doi.org/https://doi.org/10.1002/app.29845>.
